# Fish Collagen Peptides Enhance Thymopoietic Gene Expression, Cell Proliferation, Thymocyte Adherence, and Cytoprotection in Thymic Epithelial Cells via Activation of the Nuclear Factor-κB Pathway, Leading to Thymus Regeneration after Cyclophosphamide-Induced Injury

**DOI:** 10.3390/md21100531

**Published:** 2023-10-12

**Authors:** Do Young Lee, Won Hoon Song, Ye Seon Lim, Changyong Lee, Lata Rajbongshi, Seon Yeong Hwang, Byoung Soo Kim, Dongjun Lee, Yong Jung Song, Hwi-Gon Kim, Sik Yoon

**Affiliations:** 1 Department of Anatomy and Convergence Medical Sciences, Pusan National University College of Medicine, Yangsan 626-870, Republic of Korea; osldy@naver.com (D.Y.L.); yeseonlim@pusan.ac.kr (Y.S.L.); qhrrn79@naver.com (C.L.); latapharm@gmail.com (L.R.); anatomy2017@pusan.ac.kr (S.Y.H.); 2 Immune Reconstitution Research Center of Medical Research Institute, Pusan National University College of Medicine, Yangsan 626-870, Republic of Korea; luchen99@hanmail.net (W.H.S.); gynsong@gmail.com (Y.J.S.); bislsan@naver.com (H.-G.K.); 3 Department of Urology, Pusan National University Yangsan Hospital and Pusan National University College of Medicine, Yangsan 626-870, Republic of Korea; 4 School of Biomedical Convergence Engineering, Pusan National University, Yangsan 626-870, Republic of Korea; bskim7@pusan.ac.kr; 5 Department of Convergence Medicine, Pusan National University College of Medicine, Yangsan 626-870, Republic of Korea; lee.dongjun@pusan.ac.kr; 6 Department of Obstetrics and Gynecology, Pusan National University Yangsan Hospital and Pusan National University College of Medicine, Yangsan 626-870, Republic of Korea

**Keywords:** fish collagen peptides, thymus, thymic epithelial cells, thymic regeneration, thymopoietic factor, NF-κB pathway

## Abstract

Prolonged thymic involution results in decreased thymopoiesis and thymic output, leading to peripheral T-cell deficiency. Since the thymic-dependent pathway is the only means of generating fully mature T cells, the identification of strategies to enhance thymic regeneration is crucial in developing therapeutic interventions to revert immune suppression in immunocompromised patients. The present study clearly shows that fish collagen peptides (FCPs) stimulate activities of thymic epithelial cells (TECs), including cell proliferation, thymocyte adhesion, and the gene expression of thymopoietic factors such as FGF-7, IGF-1, BMP-4, VEGF-A, IL-7, IL-21, RANKL, LTβ, IL-22R, RANK, LTβR, SDF-1, CCL21, CCL25, CXCL5, Dll1, Dll4, Wnt4, CD40, CD80, CD86, ICAM-1, VCAM-1, FoxN1, leptin, cathepsin L, CK5, and CK8 through the NF-κB signal transduction pathway. Furthermore, our study also revealed the cytoprotective effects of FCPs on TECs against cyclophosphamide-induced cellular injury through the NF-κB signaling pathway. Importantly, FCPs exhibited a significant capability to facilitate thymic regeneration in mice after cyclophosphamide-induced damage via the NF-κB pathway. Taken together, this study sheds light on the role of FCPs in TEC function, thymopoiesis, and thymic regeneration, providing greater insight into the development of novel therapeutic strategies for effective thymus repopulation for numerous clinical conditions in which immune reconstitution is required.

## 1. Introduction

T cells play a central role in orchestrating the entire immune response to various pathogens like bacteria, viruses, fungi, and parasites, as well as abnormal cells, such as cancer cells. The thymus is the primary organ for T-cell development. For the development of T cells, T-cell progenitors must migrate from the bone marrow to the thymus, where immature T cells called thymocytes mature into immunocompetent T cells expressing a highly diverse antigen recognition T-cell receptor repertoire [1]. Thymopoiesis relies on multi-step processes of a complex and highly coordinated interaction between thymocytes and the thymic stromal cell microenvironment. Thymic epithelial cells (TECs) in particular represent the main stromal cell type of the thymus, providing a specialized niche that properly nurtures the developing T cells and plays key instructive roles in regulating all stages of thymopoiesis, including T-cell progenitor migration, T-cell lineage commitment, T-cell receptor (TCR) formation, CD4^+^ versus CD8^+^ lineage choice, positive selection, and negative selection [2]. Aside from communication through direct cell–cell contact, a variety of signaling molecules, such as cytokines, chemokines, and growth factors produced in the multiple thymic stromal cell types, particularly TECs, play vital roles in orchestrating intricate intercellular communication from a wide range of indispensable cellular processes, such as cell survival, division, differentiation, and movements, for the development of T cells [3,4].

Furthermore, the thymus is a unique organ that gradually degenerates with advancing age after puberty at an accelerated speed in comparison to any other tissues and organs in the body [5]. Immunosenescence is linked to this age-related thymic involution characterized by a progressive regression in the thymus size, a decreased thymic cellularity, a disrupted thymic architecture, a dramatic reduction in TECs, an expansion of adipocytes and fibroblasts, and a consequent decline in naïve T-cell output from the thymus [6,7]. Therefore, thymus involution results in decreased thymopoiesis, a diminished influx of recent thymic emigrants into the peripheral naïve T-cell pool, and a consequent reduction in the diversity of the TCR repertoire of the peripheral T cells, leading to defective immune responses against new foreign antigens [7,8]. Unexpectedly, it has been suggested that age-related thymic involution is mainly due to the age-dependent deterioration of TEC function and a reduction in the number of TECs, although scientists have long surmised that the reduced intrinsic capacity of the aged T-cell progenitors might be its primary cause [2,9]. It is noticeable that approximately 30% of all TECs are proliferating in mice during the time immediately before and after birth, whereas only approximately 5% are proliferating at the age of 4 weeks with further diminished levels thereafter, indicating that the number and proliferation rate of TECs peak during the perinatal period and then decline throughout the lifetime to the very low levels seen in the aged thymus [6].

In addition to age-related thymic involution, the thymus may abruptly undergo a transient regression known as acute thymic involution due to numerous causes, including severe stress, infection, graft-versus-host disease, pregnancy, malnutrition, steroid treatment, or cytoablative treatments such as chemotherapy, cell-depleting antibodies, and radiotherapy [10,11,12]. Acute thymic involution can elicit the death of thymocytes as well as the transient degeneration of TECs and the suppression of TEC activity by acting directly on TECs and/or indirectly through the loss of cross-talk between TECs and thymocytes, resulting in the reduced expression of thymopoietic factors critical for TEC function, maintenance, and homeostasis, such as fibroblast growth factor-7 (FGF-7), FGF-21, interleukin-7 (IL-7), IL-22 receptor (IL-22R), receptor activator of nuclear factor-κB (RANK), RANK ligand (RANKL), insulin-like growth factor-1 (IGF-1), CD40, delta-like Notch ligand 4 (Dll4), Wnt family member 4 (Wnt4), CD80, CD86, forkhead box N1 (FoxN1), leptin, and C-C motif chemokine ligand 25 (CCL25) [2,5,7,11,12,13,14]. Accordingly, either age-related thymic involution or acute thymic involution is characterized by dramatic perturbations in the TEC function, resulting in impaired thymopoiesis, reduced naïve T-cell output, peripheral T-cell depletion, and compromised host immunity in a sequential manner [2]. In this context, it is remarkable that TECs perform critical functions during regeneration after thymic injury [10]. Notably, several studies have shown that various TEC-associated molecules essential for T-cell development and production, including FGF-7, IL-7, IL-22, RANKL, IGF-1, bone morphogenic protein-4 (BMP-4), Dll4, CCL21, CCL25, and FoxN1, have apparent efficacy in promoting thymopoiesis or treating damaged thymuses, and some of these approaches have been tested in phase I or phase I/II clinical trials [2,11,12]. Thus, the development of the available therapeutic strategies that restore thymic function and enhance T-cell reconstitution after periods of thymopoietic stress or thymic involution is crucial not only for efficient immune responses against pathogens, tumor antigens, and harmful stimuli, but also for optimal responses to immunotherapy for cancer or other diseases [12].

Cyclophosphamide (CP), a cytotoxic nitrogen mustard alkylating agent, is an extensively used chemotherapeutic and immunosuppressive agent. CP, like other oxazaphosphorines, is an inert prodrug that requires bioactivation predominantly by hepatic cytochrome P450 enzymes to its major active metabolite, 4-hydroxycyclophosphamide, which is then tautomerized into aldophosphamide, which is in turn converted to other cytotoxic metabolites, such as phosphoramide mustard and acrolein [15]. CP exerts its cytotoxic effects mainly through the formation of inter-strand and intra-strand DNA cross-linkage to interfere with DNA replication and is widely used in the treatment of various forms of neoplastic diseases, such as lymphoma, multiple myeloma, leukemia, ovarian cancer, breast cancer, non-small cell lung carcinoma, neuroblastoma, sarcoma, and other solid tumors, as well as in the treatment of several autoimmune diseases, such as systemic lupus erythematosus, rheumatoid arthritis, multiple sclerosis, lupus nephritis, vasculitis, polyarteritis nodosa, myositis, granulomatosis, and Behcet’s disease [16,17]. However, despite the successful application of CP in several types of human tumors and autoimmune diseases, it also possesses a wide spectrum of toxicities [17]. Among them, immunosuppression is the major dose-limiting toxicity of CP [17].

In recent years, marine collagen peptides (MCPs) have attracted increasing interest due to their excellent biological, bioavailable, biocompatible, biodegradable, and non-toxic properties, as well as numerous beneficial health effects, such as anti-oxidant, anti-aging, anti-microbial, anti-inflammatory, neuroprotective, photoprotective, anti-hypertensive, and wound healing properties, indicating their potential to deliver a wide range of novel applications in the areas of food, cosmetic, nutraceutical, pharmaceutical, and biomedical sciences or industries [18,19]. In particular, we have recently shown that fish collagen peptides (FCPs), a common type of MCPs, protect TECs against cisplatin-induced cytotoxic and oxidative injury by inhibiting mitogen-activated protein kinase (MAPK) signal transduction pathways [20]. However, the efficacy of MCPs, including FCPs, in enhancing the thymopoietic activity of TECs and promoting thymus regeneration after acute thymic involution still remains unknown.

Thus, the aim of the present study was not only to elucidate the effect of FCPs on the thymopoietic function, proliferative activity, thymocyte-adhesive property, and cytoprotective capacity of TECs, but also to shed light on their role in the regeneration following thymic injury induced by CP. To this end, this study used a HepG2 hepatocyte culture model to generate CP metabolites as well as CP-induced acute thymic injury models for the assessment of the in vitro effects of FCPs on TECs damaged by CP and the in vivo effects of FCPs on the regeneration following CP-induced acute thymic involution.

Here, the present study shows that FCPs boost the thymopoietic function of TECs and protect TECs against CP-triggered cytotoxic injury by enhancing cell proliferation, thymocyte-adhesive and cytoprotective properties, and thymopoietic gene expression and promote regeneration following CP-induced thymic involution via activation of the nuclear factor-κB (NF-κB) signal transduction pathway.

## 2. Results

### 2.1. FCPs Stimulate Thymopoietic Gene Expression in TECs

We performed a real-time quantitative reverse transcription polymerase chain reaction (qRT-PCR) analysis to detect the expression levels of key genes associated with thymopoiesis in TECs after exposure to 0.8% FCP for 48 h to elucidate whether exposure of TECs to FCPs produces thymopoietic mediators, which play an imperative role in T-cell development.

**Figure 1:**
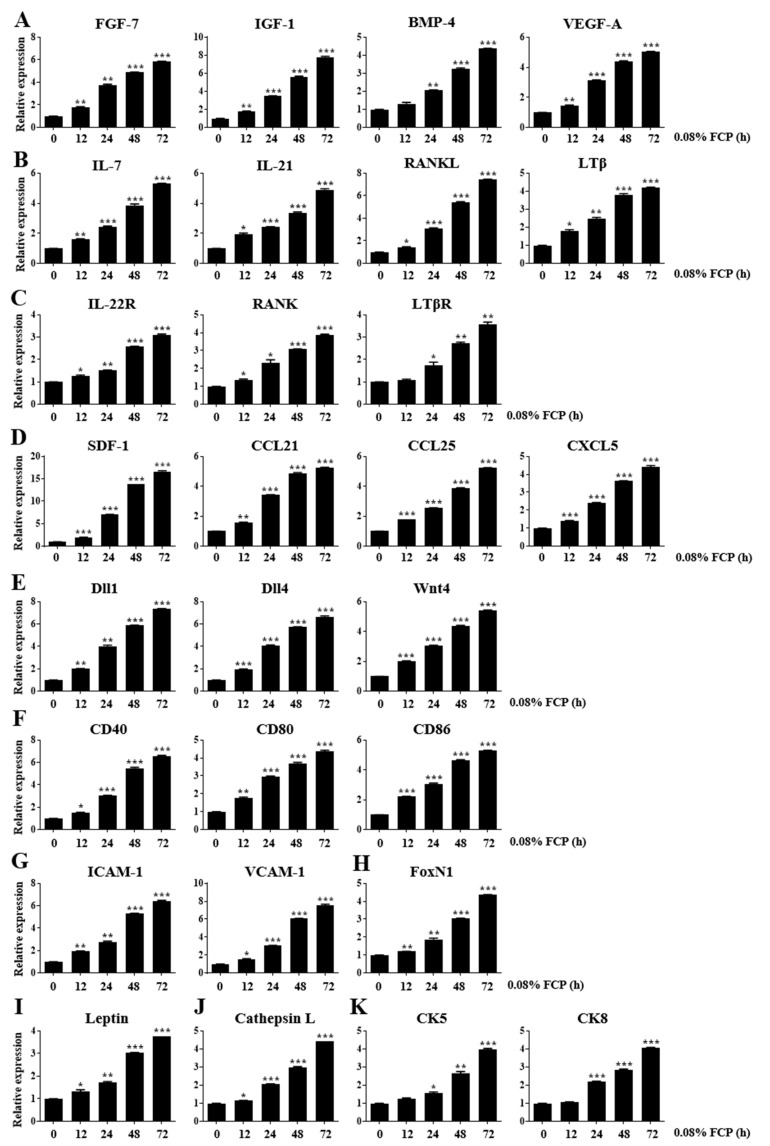
FCPs trigger thymopoietic responses in TECs as detected using qRT-PCR. Bar graphs represent the mRNA relative expression levels of (**A**) growth factors FGF-7, IGF-1, BMP-4, and VEGF-A; (**B**) thymopoietic cytokines IL-7, IL-21, RANKL, and LTβ; (**C**) thymopoietic cytokine receptors IL-22R, RANK, and LTβR; (**D**) thymopoietic chemokines SDF-1, CCL21, CCL25, and CXCL5; (**E**) thymopoietic signaling molecules Dll1, Dll4, and Wnt4; (**F**) co-stimulatory molecules CD40, CD80, and CD86; (**G**) cell adhesion molecules ICAM-1 and VCAM-1; (**H**) TEC-specific transcription factor FoxN1; (**I**) thymopoietic hormone leptin; (**J**) a protease cathepsin L; and (**K**) intermediate filaments CK5 and CK8. GAPDH was used as a housekeeping gene for normalization. All data are expressed as relative values against their respective control group. The data represent the means ± standard deviation of three independent experiments; * *p* < 0.05, ** *p* < 0.01, *** *p* < 0.001 (vs. the control group).

First, we assessed the gene expression levels of thymopoietic growth factors, such as FGF-7, IGF-1, BMP-4, and vascular endothelial growth factor-A (VEGF-A). The treatment of TECs with 0.08% FCP for 12, 24, 48, and 72 h markedly upregulated the gene expression level of FGF-7 by 1.8-fold (*p* < 0.01), 3.7-fold (*p* < 0.01), 4.9-fold (*p* < 0.001), and 5.9-fold (*p* < 0.001), respectively, in a time-dependent manner, compared with that in the control (Figure 1A). The treatment of TECs with 0.08% FCP for 12, 24, 48, and 72 h significantly enhanced the gene expression level of IGF-1 by 1.7-fold (*p* < 0.01), 3.5-fold (*p* < 0.001), 5.6-fold (*p* < 0.001), and 7.8-fold (*p* < 0.001), respectively, in a time-dependent manner, compared with that in the control (Figure 1A). The treatment of TECs with 0.08% FCP for 12, 24, 48, and 72 h significantly increased the gene expression level of BMP-4 by 1.3-fold, 2.1-fold (*p* < 0.01), 3.2-fold (*p* < 0.001), and 4.4-fold (*p* < 0.001), respectively, compared with that in the control (Figure 1A). The treatment of TECs with 0.08% FCP for 12, 24, 48, and 72 h significantly augmented the gene expression level of VEGF-A by 1.5-fold (*p* < 0.01), 3.1-fold (*p* < 0.001), 4.4-fold (*p* < 0.001), and 5.1-fold (*p* < 0.001), respectively, in a time-dependent manner, compared with that in the control (Figure 1A).

Second, we measured the gene expression levels of thymopoietic cytokines, such as IL-7, IL-21, RANKL, and lymphotoxin β (LTβ). The treatment of TECs with 0.08% FCP for 12, 24, 48, and 72 h significantly upregulated the gene expression level of IL-7 by 1.6-fold (*p* < 0.01), 2.4-fold (*p* < 0.001), 3.9-fold (*p* < 0.001), and 5.3-fold (*p* < 0.001), respectively, in a time-dependent manner, compared with that in the control (Figure 1B). The treatment of TECs with 0.08% FCP for 12, 24, 48, and 72 h significantly enhanced the gene expression level of IL-21 by 2.0-fold (*p* < 0.05), 2.4-fold (*p* < 0.001), 3.4-fold (*p* < 0.001), and 4.9-fold (*p* < 0.001), respectively, in a time-dependent manner, compared with that in the control (Figure 1B). The treatment of TECs with 0.08% FCP for 12, 24, 48, and 72 h significantly increased the gene expression level of RANKL by 1.4-fold (*p* < 0.05), 3.1-fold (*p* < 0.001), 5.4-fold (*p* < 0.001), and 7.4-fold (*p* < 0.001), respectively, in a time-dependent manner, compared with that in the control (Figure 1B). The treatment of TECs with 0.08% FCP for 12, 24, 48, and 72 h significantly augmented the gene expression level of LTβ by 1.8-fold (*p* < 0.05), 2.4-fold (*p* < 0.01), 3.8-fold (*p* < 0.001), and 4.2-fold (*p* < 0.001), respectively, in a time-dependent manner, compared with that in the control (Figure 1B).

Third, we evaluated the gene expression levels of thymopoietic cytokine receptors, such as IL-22R, RANK, and LTβ receptor (LTβR). The treatment of TECs with 0.08% FCP for 12, 24, 48, and 72 h significantly upregulated the gene expression level of IL-22R by 1.3-fold (*p* < 0.05), 1.5-fold (*p* < 0.01), 2.6-fold (*p* < 0.001), and 3.1-fold (*p* < 0.001), respectively, in a time-dependent manner, compared with that in the control (Figure 1C). The treatment of TECs with 0.08% FCP for 12, 24, 48, and 72 h significantly increased the gene expression level of RANK by 1.4-fold (*p* < 0.05), 2.3-fold (*p* < 0.05), 3.1-fold (*p* < 0.001), and 3.9-fold (*p* < 0.001), respectively, in a time-dependent manner, compared with that in the control (Figure 1C). The treatment of TECs with 0.08% FCP for 12, 24, 48, and 72 h significantly augmented the gene expression level of LTβR by 1.1-fold, 1.7-fold (*p* < 0.05), 2.7-fold (*p* < 0.01), and 3.6-fold (*p* < 0.01), respectively, in a time-dependent manner, compared with that in the control (Figure 1C).

Fourth, we determined the gene expression levels of thymopoietic chemokines, such as stromal-cell-derived factor-1 (SDF-1), CCL21, CCL25, and CXCL5. The treatment of TECs with 0.08% FCP for 12, 24, 48, and 72 h significantly upregulated the gene expression level of SDF-1 by 2.0-fold (*p* < 0.001), 7.1-fold (*p* < 0.001), 13.8-fold (*p* < 0.001), and 16.6-fold (*p* < 0.001), respectively, in a time-dependent manner, compared with that in the control (Figure 1D). The treatment of TECs with 0.08% FCP for 12, 24, 48, and 72 h significantly enhanced the gene expression level of CCL21 by 1.5-fold (*p* < 0.05), 3.0-fold (*p* < 0.001), 5.5-fold (*p* < 0.001), and 6.5-fold (*p* < 0.001), respectively, in a time-dependent manner, compared with that in the control (Figure 1D). The treatment of TECs with 0.08% FCP for 12, 24, 48, and 72 h significantly augmented the gene expression level of CCL25 by 1.8-fold (*p* < 0.001), 2.5-fold (*p* < 0.001), 3.9-fold (*p* < 0.001), and 5.2-fold (*p* < 0.001), respectively, in a time-dependent manner, compared with that in the control (Figure 1D). The treatment of TECs with 0.08% FCP for 12, 24, 48, and 72 h significantly boosted the gene expression level of CXCL5 by 1.4-fold (*p* < 0.001), 2.4-fold (*p* < 0.001), 3.6-fold (*p* < 0.001), and 4.4-fold (*p* < 0.001), respectively, in a time-dependent manner, compared with that in the control (Figure 1D).

Fifth, we examined the gene expression levels of cell signaling molecules, such as Dll1, Dll4, and Wnt4. The treatment of TECs with 0.08% FCP for 12, 24, 48, and 72 h significantly upregulated the gene expression level of Dll1 by 2.0-fold (*p* < 0.01), 4.0-fold (*p* < 0.01), 5.9-fold (*p* < 0.001), and 7.3-fold (*p* < 0.001), respectively, in a time-dependent manner, compared with that in the control (Figure 1E). The treatment of TECs with 0.08% FCP for 12, 24, 48, and 72 h significantly enhanced the gene expression level of Dll4 by 2.0-fold (*p* < 0.001), 4.1-fold (*p* < 0.001), 5.7-fold (*p* < 0.001), and 6.7-fold (*p* < 0.001), respectively, in a time-dependent manner, compared with that in the control (Figure 1E). The treatment of TECs with 0.08% FCP for 12, 24, 48, and 72 h significantly increased the gene expression level of Wnt4 by 2.0-fold (*p* < 0.001), 3.1-fold (*p* < 0.001), 4.4-fold (*p* < 0.001), and 5.4-fold (*p* < 0.001), respectively, compared with that in the control (Figure 1E).

Sixth, we investigated the gene expression levels of co-stimulatory molecules, such as CD40, CD80, and CD86. The treatment of TECs with 0.08% FCP for 12, 24, 48, and 72 h significantly increased the gene expression levels of CD40 by 1.6-fold (*p* < 0.01), 3.4-fold (*p* < 0.001), 4.9-fold (*p* < 0.001), and 5.2-fold (*p* < 0.001), respectively, in a time-dependent manner, compared with that in the control (Figure 1F). The treatment of TECs with 0.08% FCP for 12, 24, 48, and 72 h significantly upregulated the gene expression level of CD80 by 1.8-fold (*p* < 0.01), 2.9-fold (*p* < 0.001), 3.7-fold (*p* < 0.001), and 4.4-fold (*p* < 0.001), respectively, in a time-dependent manner, compared with that in the control (Figure 1F). The treatment of TECs with 0.08% FCP for 12, 24, 48, and 72 h significantly enhanced the gene expression level of CD86 by 2.2-fold (*p* < 0.001), 3.1-fold (*p* < 0.001), 4.7-fold (*p* < 0.001), and 5.3-fold (*p* < 0.001), respectively, in a time-dependent manner, compared with that in the control (Figure 1F).

Seventh, we explored the gene expression levels of cell adhesion molecules, such as intercellular cell adhesion molecule-1 (ICAM-1) and vascular cell adhesion molecule-1 (VCAM-1). The treatment of TECs with 0.08% FCP for 12, 24, 48, and 72 h significantly upregulated the gene expression level of ICAM-1 by 1.9-fold (*p* < 0.01), 2.8-fold (*p* < 0.01), 5.3-fold (*p* < 0.001), and 6.4-fold (*p* < 0.001), respectively, in a time-dependent manner, compared with that in the control (Figure 1G). The treatment of TECs with 0.08% FCP for 12, 24, 48, and 72 h significantly enhanced the gene expression level of VCAM-1 by 1.5-fold (*p* < 0.05), 3.1-fold (*p* < 0.001), 6.1-fold (*p* < 0.001), and 7.6-fold (*p* < 0.001), respectively, in a time-dependent manner, compared with that in the control (Figure 1G).

Eighth, we detected the gene expression level of FoxN1, a TEC-specific transcription factor. The treatment of TECs with 0.08% FCP for 12, 24, 48, and 72 h significantly upregulated the gene expression level of FoxN1 by 1.2-fold (*p* < 0.01), 1.9-fold (*p* < 0.01), 3.1-fold (*p* < 0.001), and 4.4-fold (*p* < 0.001), respectively, in a time-dependent manner, compared with that in the control (Figure 1H).

Ninth, we observed the gene expression level of leptin, a hormone that enhances thymopoiesis. The treatment of TECs with 0.08% FCP for 12, 24, 48, and 72 h significantly enhanced the gene expression level of leptin by 1.3-fold (*p* < 0.05), 1.7-fold (*p* < 0.05), 3.0-fold (*p* < 0.01), and 3.8-fold (*p* < 0.01), respectively, in a time-dependent manner, compared with that in the control (Figure 1I).

Tenth, we elucidated the gene expression level of the endosomal protease cathepsin L. The treatment of TECs with 0.08% FCP for 12, 24, 48, and 72 h significantly augmented the gene expression level of cathepsin L by 1.1-fold (*p* < 0.05), 2.1-fold (*p* < 0.001), 3.0-fold (*p* < 0.001), and 4.4-fold (*p* < 0.001), respectively, in a time-dependent manner, compared with that in the control (Figure 1J).

Eleventh, we checked the gene expression levels of cytokeratin (CK) intermediate filaments, such as CK5 and CK8. The treatment of TECs with 0.08% FCP for 12, 24, 48, and 72 h significantly upregulated the gene expression level of CK5 by 1.2-fold, 1.6-fold (*p* < 0.05), 2.7-fold (*p* < 0.01), and 4.0-fold (*p* < 0.001), respectively, in a time-dependent manner, compared with that in the control (Figure 1K). The treatment of TECs with FCPs for 12, 24, 48, and 72 h significantly enhanced the gene expression level of CK8 by 1.1-fold, 2.2-fold (*p* < 0.001), 2.8-fold (*p* < 0.001), and 4.1-fold (*p* < 0.001), respectively, in a time-dependent manner, compared with that in the control (Figure 1K).

### 2.2. FCPs Promote TEC Proliferation through the NF-κB Signal Transduction Pathway

**Figure 2:**
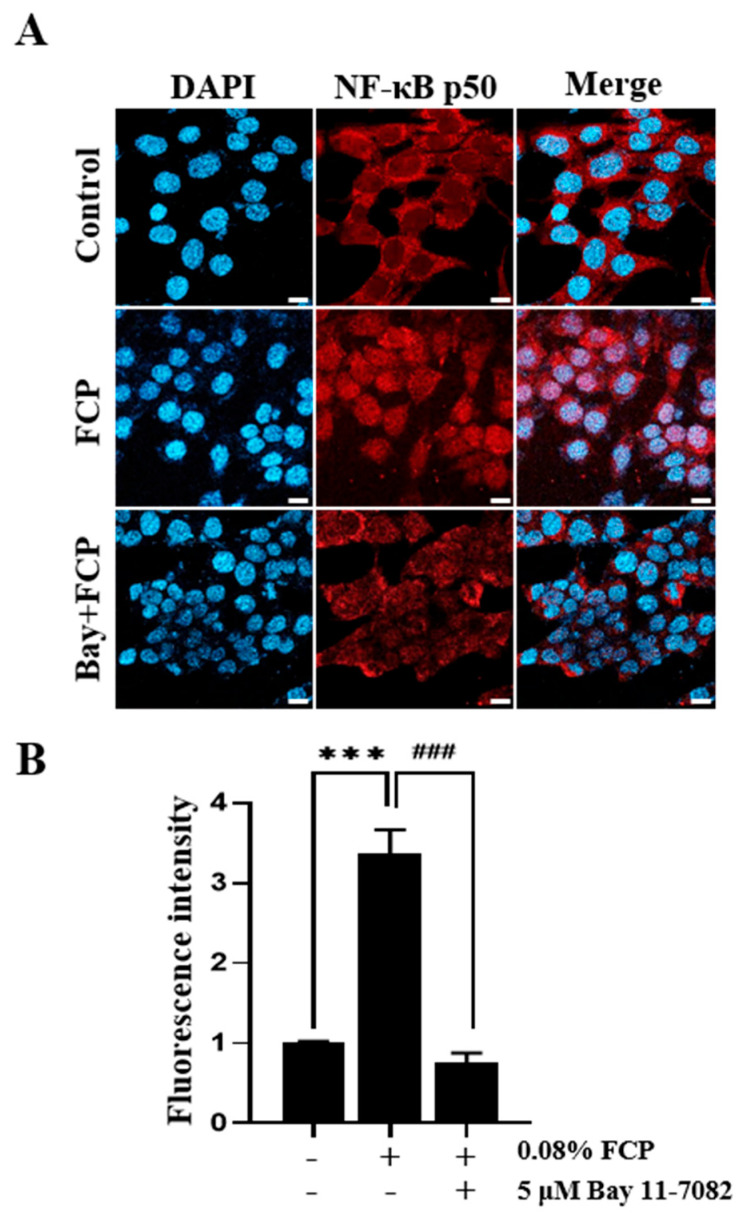
Inhibitory effects of Bay 11-7082 on the FCP-induced activation of NF-κB signaling. (**A**) Fluorescent staining and confocal laser scanning microscopy demonstrated that Bay 11-7082 inhibited the FCP-induced translocation of NF-κB p50 to the nucleus (blue: DAPI; red: NF-κB p50). (**B**) A bar graph represents the measured staining intensity of NF-κB p50 translocation. All data are expressed as relative values against their respective control group. The data represent the mean ± standard deviation of three independent experiments; *** *p* < 0.001 (vs. the control group); ^###^ *p* < 0.001 (vs. 0.08% FCP group). Scale bars = 25 µm.

As it has previously shown that adhesion with thymocytes or mature lymphoid cells can induce increased NF-κB binding activity in TECs [21,22], we first examined the nuclear translocation of NF-κB p50 in TECs by confocal microscopy in the presence and absence of Bay 11-7082 (5 µM), a selective inhibitor of NF-κB activation. FCP (0.08%) stimulation dramatically enhanced the translocation of the cytosolic NF-κB p50 to the nucleus by 3.5-fold (*p* < 0.05) compared with the control, whereas treatment with Bay 11-7082 completely blocked the FCP-induced activation of NF-κB p50, indicating that the NF-κB signaling pathway was activated by FCP exposure in TECs (Figure 2).

**Figure 3:**
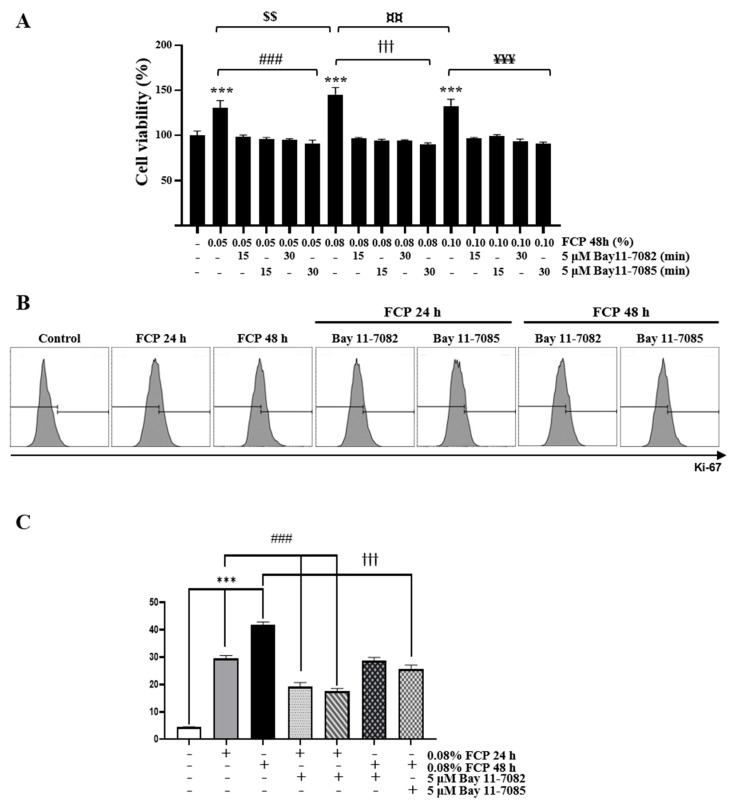
FCPs enhance the proliferation of TECs through the NF-κB signaling pathway. (**A**) The selective NF-ĸB inhibitors Bay 11-7082 and Bay 11-7085 at 5 μM inhibited cell proliferation induced by FCPs in TECs as measured by the WST-1 assay. (**B**) Flow cytometry analysis of intracellular Ki-67 expression in TECs. (**C**) A bar graph showing Ki-67 positive cell counts. The data represent the mean ± standard deviation of three independent experiments; *** *p* < 0.001 (vs. the control group); ^###^ *p* < 0.001 (vs. 0.05% FCP group); ^†††^ *p* < 0.001 (vs. 0.08% FCP group); ^¥¥¥^ *p* < 0.001 (vs. 0.1% FCP group); ^$$^ *p* < 0.01 (vs. 0.05% FCP group); ^¤¤^ *p* < 0.01 (vs. 0.08% FCP group).

Next, a CCK-8 cell proliferation assay was used to evaluate the ability of FCPs to facilitate cell proliferation. The treatment of TECs with FCPs for 48 h significantly promoted cell proliferation at concentrations of 0.05%, 0.08%, and 0.1% by 30.5% (*p* < 0.001), 45.4% (*p* < 0.001), 32.7% (*p* < 0.001), respectively, compared with the control (Figure 3A). To determine whether FCP-mediated TEC proliferation could be regulated by NF-κB signaling pathways, the TECs were stimulated with 0.05%, 0.08%, and 0.1% FCP for 48 h with or without Bay 11-7082 and Bay 11-7085. Consequently, based on the CCK-8 assay results, we found that FCP-induced cell proliferation was completely abolished by 5 µM Bay 11-7082- and Bay 11-7085-mediated NF-κB signaling inhibition (Figure 3A). In consistence with this finding, we also observed that the expression of Ki-67 in TECs by flow cytometry following incubation with 0.08% FCP for 24 and 48 h was increased by 6.7-fold (*p* < 0.001) and 9.4-fold (*p* < 0.001), respectively, whereas this FCP-induced increase in Ki-67 expression was effectively abrogated by the treatment with 5 µM of both Bay 11-7082 and Bay 11-7085 (Figure 3B). Taken together, these results confirmed that FCPs trigger cell proliferation through the NF-κB signaling pathway in TECs.

### 2.3. FCPs Enhance Thymocyte Adhesion to TECs through the NF-κB Signal Transduction Pathway

**Figure 4:**
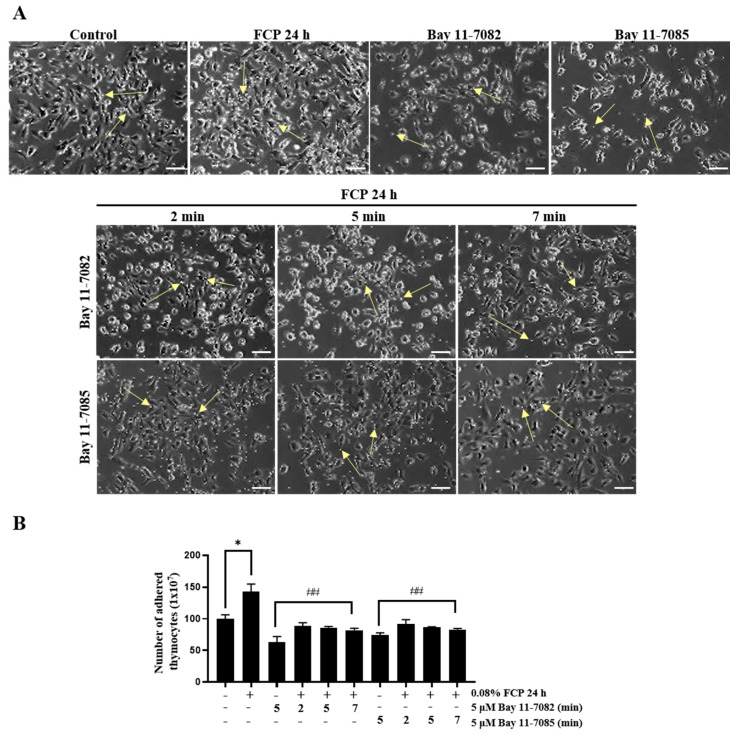
Quantitative adherence assay of thymocytes to TECs. The TECs were pretreated with 5 μM Bay 11-7082 or 11-7085 for 2, 5, and 7 min prior to seeding freshly isolated thymocytes onto the layer of TECs in 0.08% FCP-containing media for 24 h. (**A**) Representative photomicrographs of thymocytes (arrows) adhered to TECs. Treatment with 0.08% FCP for 24 h exhibited a significant increase in the number of adherent thymocytes, whereas this effect of FCPs was fully abrogated by Bay 11-7082 and Bay 11-7085. (**B**) A bar graph showing the number of adhered thymocytes. The data are expressed as the mean ± SD; * *p* < 0.05 (vs. the control group); ^##^ *p* < 0.001 (vs. 0.08% FCP group). Scale bars = 50 µm.

Thymocyte–TEC adhesion is an indispensable, fundamental biological process in T-cell development. To assess whether FCPs affect the adherence of thymocytes to TECs, a cell adhesion assay was performed. TECs treated with 0.08% FCP for 24 h exhibited a significant increase (1.4-fold) in the number of adherent thymocytes to TECs, whereas the FCP-induced stimulation of thymocyte–TEC adhesion was completely obliterated by Bay 11-7082- and Bay 11-7085-mediated NF-κB signaling inhibition (Figure 4). This finding demonstrates that FCPs promote thymocyte–TEC adhesion through the NF-κB signaling pathway.

### 2.4. FCPs Augment Thymopoietic Gene Expression in TECs through the NF-κB Signaling Pathway

**Figure 5:**
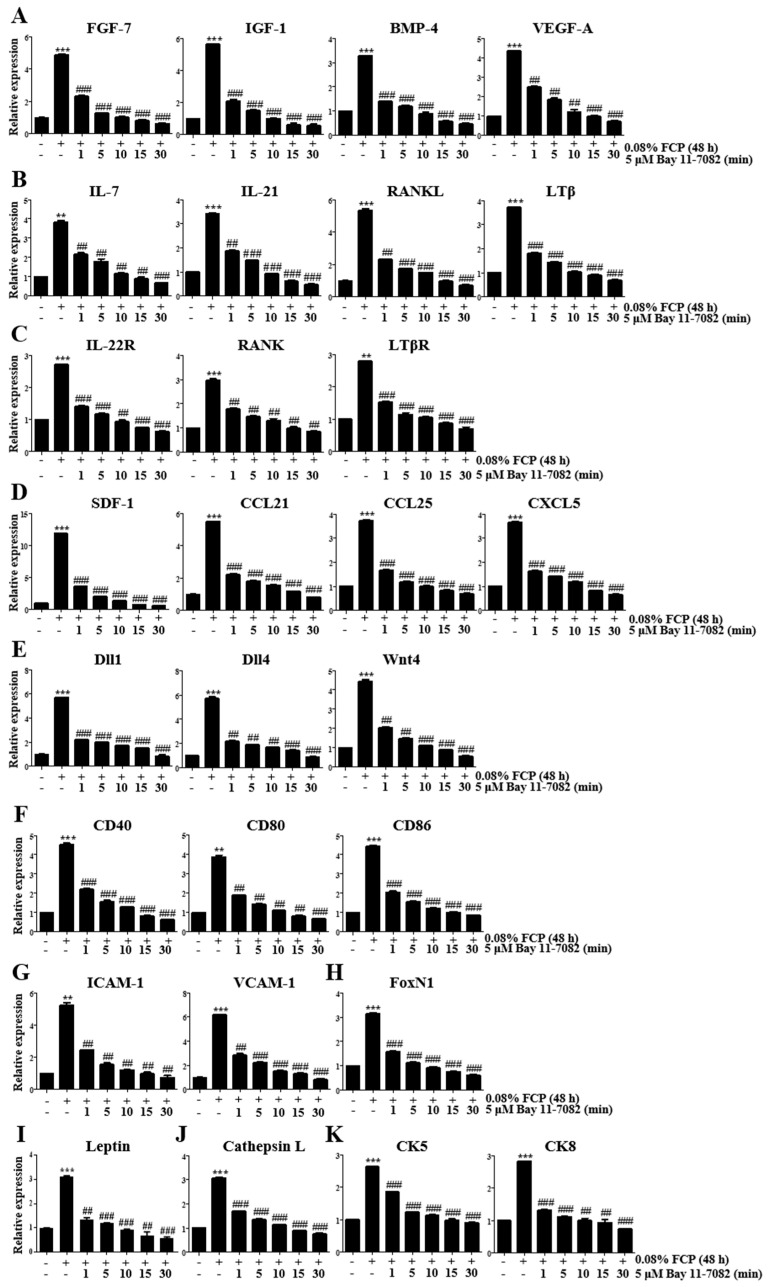
FCPs stimulate thymopoietic responses through the NF-ĸB signaling pathway in TECs as detected using qRT-PCR after treatment with Bay 11-7082. Bar graphs represent the mRNA relative expression levels of (**A**) growth factors FGF-7, IGF-1, BMP-4, and VEGF-A; (**B**) thymopoietic cytokines IL-7, IL-21, RANKL, and LTβ; (**C**) thymopoietic cytokine receptors IL-22R, RANK, and LTβR; (**D**) thymopoietic chemokines SDF-1, CCL21, CCL25, and CXCL5; (**E**) thymopoietic signaling molecules Dll1, Dll4, and Wnt4; (**F**) co-stimulatory molecules CD40, CD80, and CD86; (**G**) cell adhesion molecules ICAM-1 and VCAM-1; (**H**) TEC-specific transcription factor FoxN1; (**I**) thymopoietic hormone leptin; (**J**) a protease cathepsin L; and (**K**) intermediate filaments CK5 and CK8. GAPDH was used as a housekeeping gene for normalization. All data are expressed as relative values against their respective control group. The data represent the means ± standard deviation of three independent experiments; ** *p* < 0.01 (vs. the control group), *** *p* < 0.001 (vs. the control group); ^##^ *p* < 0.01 (vs. 0.08% FCP group), ^###^ *p* < 0.001 (vs. 0.08% FCP group).

To determine whether the FCP-induced generation of thymopoietic factors requires activation of the NF-κB signaling pathway, the expression of thymopoietic factors was evaluated in TECs using a qRT-PCR assay after treatment with 0.08% FCP in the presence or absence of 5 μM Bay 11-7082 (Figure 5) and Bay 11-7085 (Figure S1). Remarkably, it was verified that the FCP-induced upregulation of FGF-7, IGF-1, BMP-4, VEGF-A, IL-7, IL-21, RANKL, LTβ, IL-22R, RANK, LTβR, SDF-1, CCL21, CCL25, CXCL5, Dll1, Dll4, Wnt4, CD40, CD80, CD86, ICAM-1, VCAM-1, FoxN1, leptin, cathepsin L, CK5, and CK8 gene expression was robustly abolished by Bay 11-7082 and Bay 11-7085, indicating that FCPs trigger the production of thymopoietic factors in TECs through the NF-κB signal transduction pathway (Figure 5 and Figure S1).

### 2.5. FCPs Protect TECs against CP-Induced Cellular Injury through the NF-κB Signaling Pathway

An in vitro model of TEC injury was developed to assess the effects of FCPs on the regulation of functional recovery following CP-induced cellular damage. Using this model, we performed a qRT-PCR analysis to detect the expression levels of the key genes associated with thymopoiesis in TECs after exposure to 0.8% FCP for 48 h.

**Figure 6:**
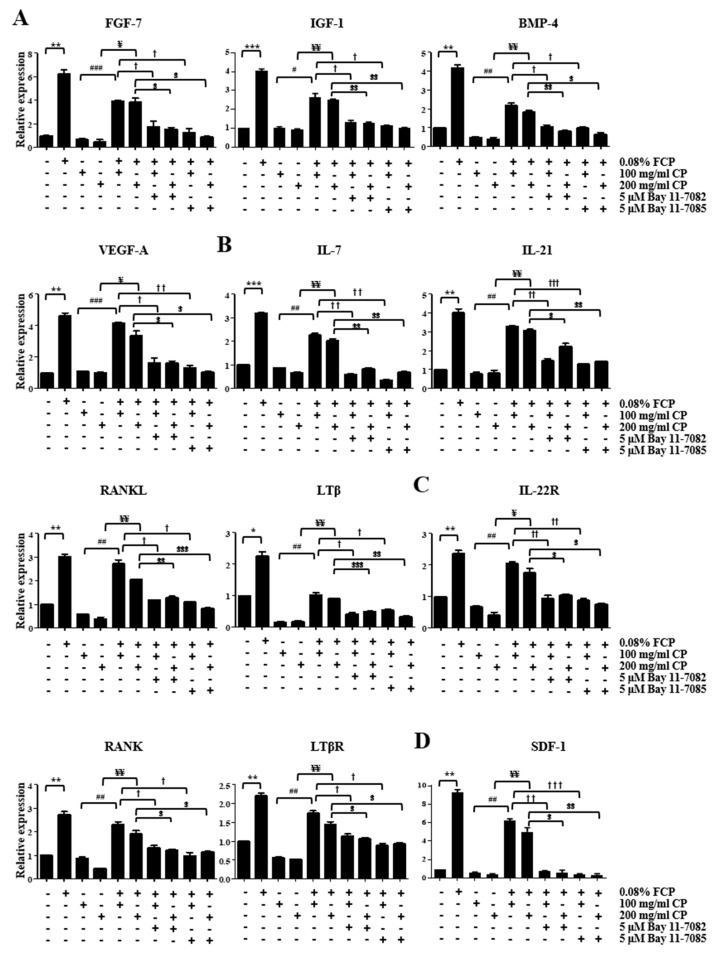

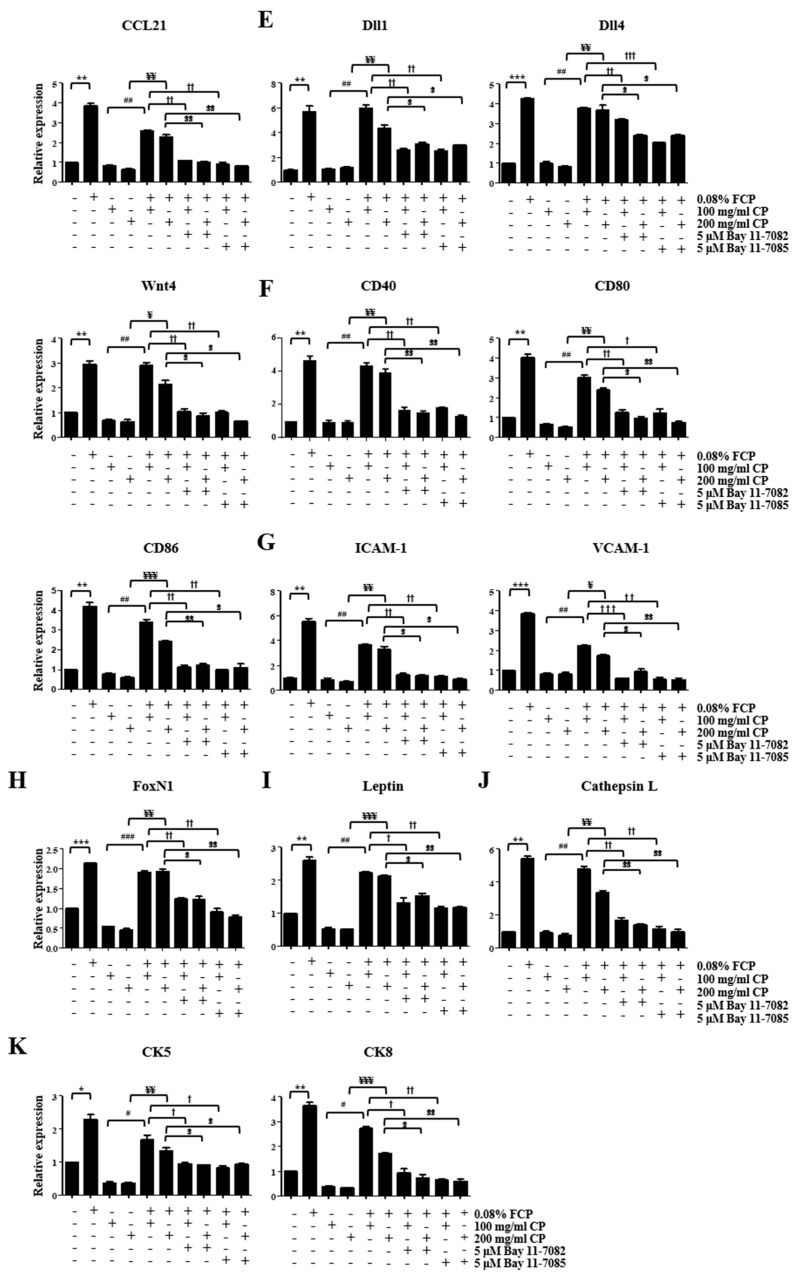
Protective effects of FCP against CP-induced cytotoxicity in TECs as detected using qRT-PCR. Bar graphs display the mRNA relative expression levels of (**A**) growth factors FGF-7, IGF-1, BMP-4, and VEGF-A; (**B**) thymopoietic cytokines IL-7, IL-21, RANKL, and LTβ; (**C**) thymopoietic cytokine receptors IL-22R, RANK, and LTβR; (**D**) thymopoietic chemokines SDF-1 and CCL21; (**E**) thymopoietic signaling molecules Dll1, Dll4, and Wnt4; (**F**) co-stimulatory molecules CD40, CD80, and CD86; (**G**) cell adhesion molecules ICAM-1 and VCAM-1; (**H**) TEC-specific transcription factor FoxN1; (**I**) thymopoietic hormone leptin; (**J**) a protease cathepsin L; and (**K**) intermediate filaments CK5 and CK8. GAPDH was used as a housekeeping gene for normalization. All data are expressed as relative values against their respective control group. The data represent the means ± standard deviation of three independent experiments; * *p* < 0.05, ** *p* < 0.01, *** *p* < 0.001 (vs. the control group); ^#^ *p* < 0.05, ^##^ *p* < 0.01, ^###^ *p* < 0.001 (vs. 100 mg/mL CP group); ^¥^ *p* < 0.05, ^¥¥^ *p* < 0.01, ^¥¥¥^ *p* < 0.001 (vs. 200 mg/mL CP group); ^†^ *p* < 0.05, ^††^ *p* < 0.01, ^†††^ *p* < 0.001 (vs. 0.08% FCP+100 mg/mL CP group); ^$^ *p* < 0.05, ^$$^ *p* < 0.01, ^$$$^ *p* < 0.001 (vs. 0.08% FCP+200 mg/mL CP group).

The treatment of CP (100 and 200 mg/mL)-pretreated TECs with 0.08% FCP for 48 h increased the FGF-7 gene expression by 5.8-fold (*p* < 0.001) and 8.5-fold (*p* < 0.05), respectively, compared with that in the CP-treated group (Figure 6A). This upregulated FGF-7 gene expression in the CP and FCP co-treated group was fully reversed by the addition of Bay 11-7082 and Bay 11-7085 (Figure 6A). The treatment of CP (100 and 200 mg/mL)-pretreated TECs with 0.08% FCP for 48 h increased IGF-1 gene expression by 2.6-fold (*p* < 0.05) and 2.7-fold (*p* < 0.01), respectively, compared with that in the CP-treated group (Figure 6A). This upregulated IGF-1 gene expression in the CP and FCP co-treated group was fully abrogated by the addition of Bay 11-7082 and Bay 11-7085 (Figure 6A). The treatment of CP (100 and 200 mg/mL)-pretreated TECs with 0.08% FCP for 48 h increased BMP-4 gene expression by 4.5-fold (*p* < 0.01) and 4.6-fold (*p* < 0.01), respectively, compared with that in the CP-treated group (Figure 6A). This upregulated BMP-4 gene expression in the CP and FCP co-treated group was fully obliterated by the addition of Bay 11-7082 and Bay 11-7085 (Figure 6A). The treatment of CP (100 and 200 mg/mL)-pretreated TECs with 0.08% FCP for 48 h increased VEGF-A gene expression by 3.9-fold (*p* < 0.001) and 3.4-fold (*p* < 0.05), respectively, compared with that in the CP-treated group (Figure 1). This upregulated VEGF-A gene expression in the CP and FCP co-treated group was fully abolished by the addition of Bay 11-7082 and Bay 11-7085 (Figure 6A).

The treatment of CP (100 and 200 mg/mL)-pretreated TECs with 0.08% FCP for 48 h increased the IL-7 gene expression by 2.6-fold (*p* < 0.01) and 3.0-fold (*p* < 0.01), respectively, compared with that in the CP-treated group (Figure 6B). This upregulated IL-7 gene expression in the CP and FCP co-treated group was fully reversed by the addition of Bay 11-7082 and Bay 11-7085 (Figure 6B). The treatment of CP (100 and 200 mg/mL)-pretreated TECs with 0.08% FCP for 48 h increased the IL-21 gene expression by 4.3-fold (*p* < 0.01) and 3.8-fold (*p* < 0.01), respectively, compared with that in the CP-treated group (Figure 6B). This upregulated IL-21 gene expression in the CP and FCP co-treated group was fully abrogated by the addition of Bay 11-7082 and Bay 11-7085 (Figure 6B). The treatment of CP (100 and 200 mg/mL)-pretreated TECs with 0.08% FCP for 48 h increased the RANKL gene expression by 4.6-fold (*p* < 0.01) and 5.4-fold (*p* < 0.01), respectively, compared with that in the CP-treated group (Figure 6B). This upregulated RANKL gene expression in the CP and FCP co-treated group was fully obliterated by the addition of Bay 11-7082 and Bay 11-7085 (Figure 6B). The treatment of CP (100 and 200 mg/mL)-pretreated TECs with 0.08% FCP for 48 h increased LTβ gene expression by 6.5-fold (*p* < 0.01) and 5.4-fold (*p* < 0.01), respectively, compared with that in the CP-treated group (Figure 6B). This upregulated LTβ gene expression in the CP and FCP co-treated group was fully abolished by the addition of Bay 11-7082 and Bay 11-7085 (Figure 6B).

The treatment of CP (100 and 200 mg/mL)-pretreated TECs with 0.08% FCP for 48 h increased the IL-22R gene expression by 3.0-fold (*p* < 0.01) and 4.4-fold (*p* < 0.05), respectively, compared with that in the CP-treated group (Figure 6C). This upregulated IL-22R gene expression in the CP and FCP co-treated group was fully reversed by the addition of Bay 11-7082 and Bay 11-7085 (Figure 6C). The treatment of CP (100 and 200 mg/mL)-pretreated TECs with 0.08% FCP for 48 h increased the RANK gene expression by 2.6-fold (*p* < 0.01) and 4.4-fold (*p* < 0.01), respectively, compared with that in the CP-treated control (Figure 6C). This upregulated RANK gene expression in the CP and FCP co-treated group was fully obliterated by the addition of Bay 11-7082 and Bay 11-7085 (Figure 6C). The treatment of CP (100 and 200 mg/mL)-pretreated TECs with 0.08% FCP for 48 h increased the LTβR gene expression by 3.1-fold (*p* < 0.01) and 2.8-fold (*p* < 0.01), respectively, compared with that in the CP-treated group (Figure 6C). This upregulated LTβR gene expression in the CP and FCP co-treated group was fully abolished by the addition of Bay 11-7082 and Bay 11-7085.

The treatment of CP (100 and 200 mg/mL)-pretreated TECs with 0.08% FCP for 48 h increased SDF-1 gene expression by 11.1-fold (*p* < 0.01) and 11.1-fold (*p* < 0.01), respectively, compared with that in the CP-treated group (Figure 6D). This upregulated SDF-1 gene expression in the CP and FCP co-treated group was fully reversed by the addition of Bay 11-7082 and Bay 11-7085 (Figure 6D). The treatment of CP (100 and 200 mg/mL)-pretreated TECs with 0.08% FCP for 48 h increased CCL21 gene expression by 3.1-fold (*p* < 0.01) and 3.6-fold (*p* < 0.01), respectively, compared with that in the CP-treated group (Figure 6D). This upregulated CCL21 gene expression in the CP and FCP co-treated group was fully abrogated by the addition of Bay 11-7082 and Bay 11-7085 (Figure 6D).

The treatment of CP (100 and 200 mg/mL)-pretreated TECs with 0.08% FCP for 48 h increased Dll1 gene expression by 5.6-fold (*p* < 0.01) and 3.8-fold (*p* < 0.01), respectively, compared with that in the CP-treated control (Figure 6E). This upregulated Dll1 gene expression in the CP and FCP co-treated group was fully reversed by the addition of Bay 11-7082 and Bay 11-7085 (Figure 6E). The treatment of CP (100 and 200 mg/mL)-pretreated TECs with 0.08% FCP for 48 h increased Dll4 gene expression by 3.8-fold (*p* < 0.01) and 4.4-fold (*p* < 0.01), respectively, compared with that in the CP-treated group (Figure 6E). This upregulated Dll4 gene expression in the CP and FCP co-treated group was fully abrogated by the addition of Bay 11-7082 and Bay 11-7085 (Figure 6E). Treatment of CP (100 and 200 mg/mL)-pretreated TECs with 0.08% FCP for 48 h increased Wnt4 gene expression by 4.3-fold (*p* < 0.01) and 3.4-fold (*p* < 0.05), respectively, compared with that in the CP-treated control (Figure 6E). This upregulated Wnt4 gene expression in the CP and FCP co-treated group was fully abolished by the addition of Bay 11-7082 and Bay 11-7085 (Figure 6E).

The treatment of CP (100 and 200 mg/mL)-pretreated TECs with 0.08% FCP for 48 h increased CD40 gene expression by 4.7-fold (*p* < 0.01) and 4.3-fold (*p* < 0.01), respectively, compared with that in the CP-treated group (Figure 6F). This upregulated CD40 gene expression in the CP and FCP co-treated group was fully obliterated by the addition of Bay 11-7082 and Bay 11-7085 (Figure 6F). The treatment of CP (100 and 200 mg/mL)-pretreated TECs with 0.08% FCP for 48 h increased CD80 gene expression by 4.6-fold (*p* < 0.01) and 4.5-fold (*p* < 0.01), respectively, compared with that in the CP-treated group (Figure 6F). This upregulated CD80 gene expression in the CP and FCP co-treated group was fully reversed by the addition of Bay 11-7082 and Bay 11-7085 (Figure 6F). The treatment of CP (100 and 200 mg/mL)-pretreated TECs with 0.08% FCP for 48 h increased CD86 gene expression by 4.4-fold (*p* < 0.01) and 4.1-fold (*p* < 0.001), respectively, compared with that in the CP-treated control (Figure 6F). This upregulated CD86 gene expression in the CP and FCP co-treated group was fully abrogated by the addition of Bay 11-7082 and Bay 11-7085 (Figure 6F).

The treatment of CP (100 and 200 mg/mL)-pretreated TECs with 0.08% FCP for 48 h increased ICAM-1 gene expression by 4.3-fold (*p* < 0.01) and 4.7-fold (*p* < 0.01), respectively, compared with that in the CP-treated group (Figure 6G). This upregulated ICAM-1 gene expression in the CP and FCP co-treated group was fully reversed by the addition of Bay 11-7082 and Bay 11-7085 (Figure 6G). The treatment of CP (100 and 200 mg/mL)-pretreated TECs with 0.08% FCP for 48 h increased VCAM-1 gene expression by 2.8-fold (*p* < 0.01) and 2.1-fold (*p* < 0.05), respectively, compared with that in the CP-treated group (Figure 6G). This upregulated VCAM-1 gene expression in the CP and FCP co-treated group was fully abrogated by the addition of Bay 11-7082 and Bay 11-7085 (Figure 6G).

The treatment of CP (100 and 200 mg/mL)-pretreated TECs with 0.08% FCP for 48 h increased FoxN1 gene expression by 3.5-fold (*p* < 0.001) and 4.2-fold (*p* < 0.01), respectively, compared with that in the CP-treated group (Figure 6H). This upregulated FoxN1 gene expression in the CP and FCP co-treated group was fully reversed by the addition of Bay 11-7082 and Bay 11-7085 (Figure 6H).

The treatment of CP (100 and 200 mg/mL)-pretreated TECs with 0.08% FCP for 48 h increased leptin gene expression by 4.2-fold (*p* < 0.01) and 4.1-fold (*p* < 0.001), respectively, compared with that in the CP-treated group (Figure 6I). This upregulated leptin gene expression in the CP and FCP co-treated group was fully abrogated by the addition of Bay 11-7082 and Bay 11-7085 (Figure 6I).

The treatment of CP (100 and 200 mg/mL)-pretreated TECs with 0.08% FCP for 48 h increased cathepsin L gene expression by 5.2-fold (*p* < 0.01) and 4.3-fold (*p* < 0.01), respectively, compared with that in the CP-treated group (Figure 6J). This upregulated cathepsin L gene expression in the CP and FCP co-treated group was fully obliterated by the addition of Bay 11-7082 and Bay 11-7085 (Figure 6J).

The treatment of CP (100 and 200 mg/mL)-pretreated TECs with 0.08% FCP for 48 h increased CK5 gene expression by 4.6-fold (*p* < 0.05) and 3.6-fold (*p* < 0.01), respectively, compared with that in the CP-treated control (Figure 6K). This upregulated CK5 gene expression in the CP and FCP co-treated group was fully abolished by the addition of Bay 11-7082 and Bay 11-7085 (Figure 6K). The treatment of CP (100 and 200 mg/mL)-pretreated TECs with 0.08% FCP for 48 h increased CK8 gene expression by 7.3-fold (*p* < 0.01) and 5.3-fold (*p* < 0.001), respectively, compared with that in the CP-treated group (Figure 6K). This upregulated CK8 gene expression in the CP and FCP co-treated group was fully revoked by the addition of Bay 11-7082 and Bay 11-7085 (Figure 6K).

### 2.6. FCPs Promote Regeneration In Vivo from CP-Induced Thymus Injury through the NF-κB Signaling Pathway

**Figure 7:**
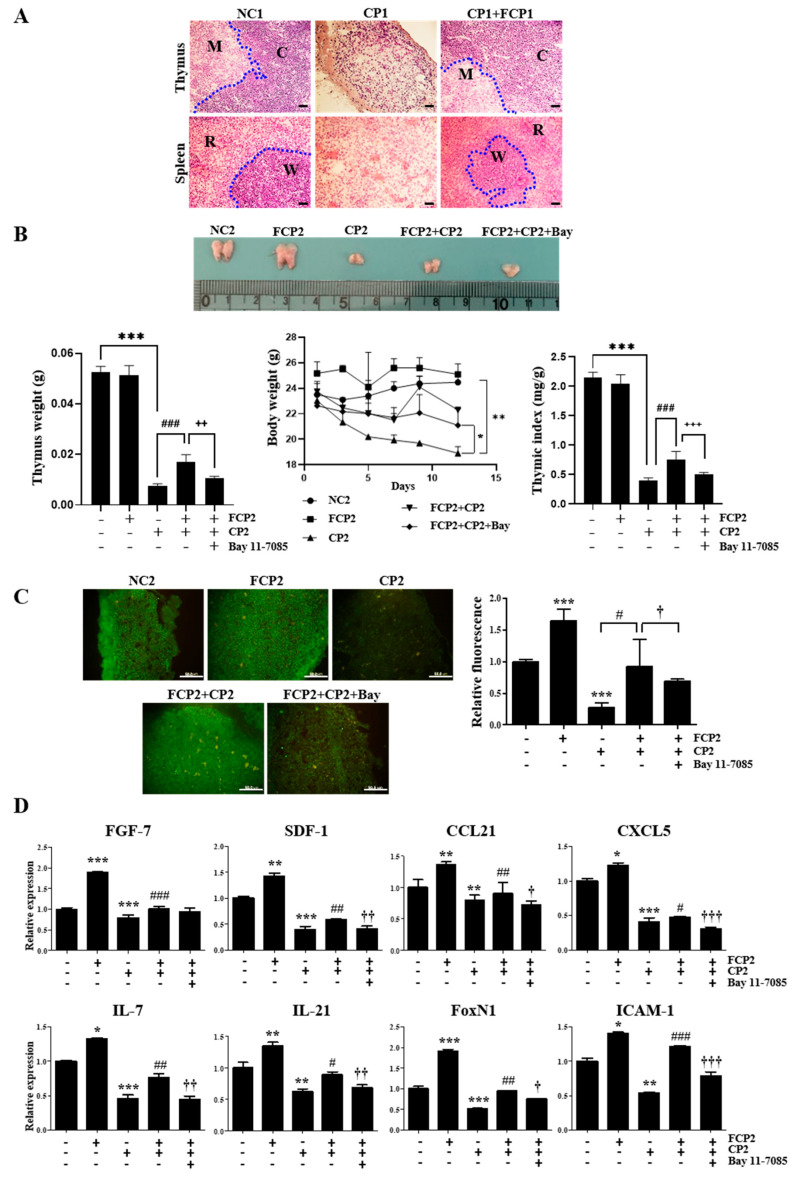
Protective effect of FCPs against CP-induced injury in mice model. (**A**) Representative HE staining of the thymus and spleen sections from the NC1, CP1, and FCP1+CP1 groups. C, cortex; M, medulla; R, red pulp; W, white pulp. (**B**) The thymus size, mice body weight, and thymic index. (**C**) Representative immunofluorescence staining of thymus sections to detect CD4 expression (green), and (**D**) bar graphs represent the mRNA relative expression levels of FGF-7, SDF-1, CCL21, CXCL5, IL-7, IL-21, FoxN1, and ICAM-1, normalized to GAPDH, from NC2, FCP2, CP2, FCP2+CP2, and FCP2+CP2+Bay groups. The data shown are means ± standard deviation; * *p* < 0.05, ** *p* < 0.01, *** *p* < 0.001 (vs. the NC2 group); ^#^ *p* < 0.05, ^##^ *p* < 0.01, ^###^ *p* < 0.001 (vs. the CP2 group); ^†^ *p* < 0.05, ^††^ *p* < 0.01, ^†††^ *p* < 0.001 (vs. FCP2+CP2 group). Scale bars = 50 µm (both in (**A**) and (**C**)).

Next, we assessed the in vivo immune-enhancing efficacy of FCPs using a histological and immunohistochemical analysis to elucidate whether FCPs can promote functional recovery following CP-induced thymus damage in mice via the NF-κB signaling pathway. In the present study, the histomorphology of the thymus and spleen was examined with a light microscope. The thymus and spleen of the normal control 1 (NC1) group displayed massive closely arranged and deeply stained thymocytes and splenocytes with an obvious nucleus (Figure 7A). Two histologically discrete compartments, the cortex and medulla in the normal thymus, and the white pulp and red pulp in the normal spleen, were clearly visible (Figure 7A). In the CP1 group, we observed a profound decrease in the number of thymocytes and splenocytes, while in the FCP1+CP1 group, the thymus and spleen cells were arranged compactly in good order with clear nuclei and less intercellular space, which were similar to those in the NC1 group (Figure 7A). These results indicate that FCPs significantly prevented damage to the thymus and spleen in the mice induced by CP.

The effects of FCPs on the gross appearance of the thymus, thymus and body weights, and thymus index are shown in Figure 7B, respectively. In the CP2 group, the thymus and body weights decreased gradually during the experimental period (Figure 7B). On day 12, the thymus and body weights and thymus index in the CP2 group were significantly reduced by approximately 7.0-fold (*p* < 0.001), 1.3-fold (*p* < 0.01), and 5.4-fold (*p* < 0.001), respectively, compared to the NC2 group (Figure 7B). In contrast, in the FCP2 group, the reduction in the thymus and body weights and thymus index caused by CP significantly improved in mice administered FCPs (Figure 7B). The thymus and body weights and thymus index of the FCP2+CP2 group increased by approximately 2.3-fold (*p* < 0.001), 1.2-fold (*p* < 0.05), and 1.9-fold (*p* < 0.001), respectively, compared to the CP2 group (Figure 7B). Notably, these increased responses in the FCP2+CP2 group were fully reversed by the administration of Bay 11-7085 (Figure 7B). These results indicate that the promoting effects of FCPs on regeneration following damage to the thymus in the mice induced by CP are mediated via the NF-κB signaling pathway.

To further analyze the effect of FCPs on the thymus, we performed anti-CD4 immunofluorescence microscopy (Figure 7C). The thymus of the NC2 and FCP2 groups contained densely packed CD4^+^ thymocytes, whereas the CP2 group resulted in almost complete disappearance of the CD4^+^ thymocytes (Figure 7C). On day 12, in the CP2 group, the fluorescence intensity of CD4^+^ thymocytes was significantly reduced by approximately 3.7-fold compared to the NC2 group (*p* < 0.001) (Figure 7C). Remarkably, in the FCP2+CP2 group, the reduction in the fluorescence intensity of CD4^+^ thymocytes caused by CP significantly improved in mice administered FCPs by approximately 3.3-fold compared to the CP2 group (*p* < 0.05), whereas the FCP-induced increase in CD4^+^ expression was completely abrogated by Bay 11-7085 in the FCP2+CP2+Bay group (Figure 7C). These results indicate that the regeneration-promoting effects of FCPs on the CD4^+^ thymocytes following acute thymic involution in the mice induced by CP was mediated via the NF-κB signaling pathway.

Furthermore, we performed a qRT-PCR analysis to measure the expression levels of key genes associated with thymopoiesis for the assessment of the effects of FCPs on the regulation of functional recovery following CP-induced cellular damage using an in vivo thymic involution and regeneration model.

First, the administration of FCPs robustly elevated the level of FGF-7 gene expression by 1.9-fold in the FCP2 group compared with the NC2 group (*p* < 0.001), whereas in the CP2 group, CP administration significantly reduced the level of FGF-7 gene expression compared with the NC2 group (Figure 7D). In the FCP2+CP2 group, the treatment of CP-pretreated mice with FCPs increased FGF-7 gene expression by 1.3-fold (*p* < 0.001) compared with that in the CP2 group (Figure 7D). This upregulated FGF-7 gene expression in the FCP2+CP2 group was reversed by the treatment with Bay 11-7085 (Figure 7D).

Second, the administration of FCPs robustly elevated the level of SDF-1 gene expression by 1.4-fold in the FCP2 group compared with the NC2 group (*p* < 0.01), whereas CP administration significantly reduced the level of SDF-1 gene expression compared with the NC2 group (Figure 7D). In the FCP2+CP2 group, the treatment of CP-pretreated mice with FCPs increased SDF-1 gene expression by 1.5-fold (*p* < 0.01) compared with that in the CP2 group (Figure 7D). This upregulated SDF-1 gene expression in the FCP2+CP2 group was reversed by the treatment with Bay 11-7085 (Figure 7D).

Third, the administration of FCPs robustly elevated the level of CCL21 gene expression by 1.4-fold in the FCP2 group compared with the NC2 group (*p* < 0.01), whereas in the CP2 group, CP administration significantly reduced the level of CCL21 gene expression compared with the NC2 group (Figure 7D). In the FCP2+CP2 group, the treatment of CP-pretreated mice with FCPs increased CCL21 gene expression by 1.1-fold (*p* < 0.01) compared with that in the CP2 group (Figure 7D). This upregulated CCL21 gene expression in the FCP2+CP2 group was reversed by treatment with Bay 11-7085 (Figure 7D).

Fourth, the administration of FCPs robustly elevated the level of CXCL5 gene expression by 1.2-fold in the FCP2 group compared with the NC2 group (*p* < 0.01), whereas in the CP2 group, CP administration significantly reduced the level of CXCL5 gene expression compared with the NC2 group (Figure 7D). In the FCP2+CP2 group, the treatment of CP-pretreated mice with FCPs increased CXCL5 gene expression by 1.2-fold (*p* < 0.01) compared with that in the CP2 group (Figure 7D). This upregulated CXCL5 gene expression in the FCP2+CP2 group was reversed with the treatment with Bay 11-7085 (Figure 7D).

Fifth, the administration of FCPs robustly elevated the level of IL-7 gene expression by 1.3-fold in the FCP2 group compared with the NC2 group (*p* < 0.01), whereas in the CP2 group, CP administration significantly reduced the level of IL-7 gene expression compared with the NC2 group (Figure 7D). In the FCP2+CP2 group, the treatment of CP-pretreated mice with FCPs increased IL-7 gene expression by 1.7-fold (*p* < 0.01) compared with that in the CP2 group (Figure 7D). This upregulated IL-7 gene expression in the FCP2+CP2 group was reversed by the treatment with Bay 11-7085 (Figure 7D).

Sixth, the administration of FCPs robustly elevated the level of IL-21 gene expression by 1.4-fold in the FCP2 group compared with the NC2 group (*p* < 0.01), whereas in the CP2 group, CP administration significantly reduced the level of IL-21 gene expression compared with the NC2 group (Figure 7D). In the FCP2+CP2 group, the treatment of CP-pretreated mice with FCPs increased IL-21 gene expression by 1.4-fold (*p* < 0.01) compared with that in the CP2 group (Figure 7D). This upregulated IL-21 gene expression in the FCP2+CP2 group was reversed by the treatment with Bay 11-7085 (Figure 7D).

Seventh, the administration of FCPs robustly elevated the level of FoxN1 gene expression by 1.9-fold in the FCP2 group compared with the NC2 group (*p* < 0.01), whereas in the CP2 group, CP administration significantly reduced the level of FoxN1 gene expression compared with the NC2 group (Figure 7D). In the FCP2+CP2 group, the treatment of CP-pretreated mice with FCPs increased FoxN1gene expression by 1.8-fold (*p* < 0.01) compared with that in the CP2 group (Figure 7D). This upregulated FoxN1 gene expression in the FCP2+CP2 group was reversed by the treatment with Bay 11-7085 (Figure 7D).

Eighth, the administration of FCPs robustly elevated the level of ICAM-1 gene expression by 1.4-fold in the FCP2 group compared with the NC2 group (*p* < 0.01), whereas in the CP2 group, CP administration significantly reduced the level of ICAM-1 gene expression compared with the NC2 group (Figure 7D). In the FCP2+CP2 group, the treatment of CP-pretreated mice with FCPs increased ICAM-1 gene expression by 2.3-fold (*p* < 0.01) compared with that in the CP2 group (Figure 7D). This upregulated ICAM-1 gene expression in the FCP2+CP2 group was reversed by the treatment with Bay 11-7085 (Figure 7D).

## 3. Discussion

We have demonstrated for the first time that FCPs enhance the proliferation, thymocyte adhesion, and thymopoietic activities of TECs, which play key roles in T-cell development and regeneration, through the NF-κB signaling pathway. Furthermore, this study revealed that FCPs not only protect TECs against CP-induced cellular injury but also facilitate thymic regeneration in mice after CP-induced damage via the NF-κB pathway. Notably, it was also found that NF-κB plays an essential role in the regulation of the FCP-inducible gene expression of a variety of thymopoietic factors, such as FGF-7, IGF-1, BMP-4, VEGF-A, IL-7, IL-21, RANKL, LTβ, IL-22R, RANK, LTβR, SDF-1, CCL21, CCL25, CXCL5, Dll1, Dll4, Wnt4, CD40, CD80, CD86, ICAM-1, VCAM-1, FoxN1, leptin, cathepsin L, CK5, and CK8. Thus, our results highlight the potential benefits of FCPs in developing therapeutic strategies to restore thymic function and enhance T-cell reconstitution after thymopoietic stress due to thymic damage or involution, especially in several clinical conditions associated with T-cell deficiency.

The thymic microenvironment plays a crucial role in the regulation of thymopoiesis through a highly coordinated and complex network of cellular and cytokine interactions that comprises thymocytes, thymic stromal cells (e.g., TECs), thymopoietic factors (e.g., growth factors, cytokines, chemokines, signaling molecules, cell adhesion molecules, co-stimulatory molecules, hormones, and transcription factors), extracellular matrix (ECM), and other soluble proteins [23]. Particularly, the thymic epithelial meshwork, the major component of the thymic microenvironment, fulfills a crucial role during specific stages of T-cell survival, proliferation, and differentiation via interactions with thymocytes, which are highly dynamic processes basically consisting of (1) thymocyte–TEC contact, (2) the production of autocrine and paracrine thymopoietic factors, and (3) signaling by direct and indirect cell–cell communications [2,3,4].

In this study, we aimed to provide a comprehensive understanding of the characteristics of thymopoietic gene expression induced by FCPs in both normal and injured TECs, and consequently, our results highlighted the impact of FCPs on thymopoietic gene expression response to TEC injury.

First, we demonstrated that FCPs significantly promote the gene expression levels of FGF-7, IGF-1, BMP-4, and VEGF-A, which are essential growth factors that mediate both prenatal and postnatal T-cell production in the thymus in an autocrine/paracrine/endocrine manner in both normal and injured TECs.

FGF-7 (also known as keratinocyte growth factor, KGF) is a potent epithelial cell-specific growth factor [24]. FGF-7 binds to and activates a specific receptor tyrosine kinase, the IIIb variant of the FgfR2 receptor (FGFR2-IIIb), which is expressed within the thymus exclusively in TECs [25]. FGF-7 enhances postnatal T-cell development via enhancements in the proliferation and function of TECs [26]. FGF-7 also plays a role in the protection of TECs from cytotoxic therapy-induced damage as well as post-BMT immune deficiency caused by the loss of TECs [27]. FGF-7 exerts a critical role in TEC differentiation [28]. Furthermore, FGF-7 promotes thymus regeneration from thymic involution in aged mice by directly stimulating TEC proliferation and differentiation [26,29,30,31].

IGF-1, a family of neuroendocrine factors, has multiple anabolic functions including increased cell proliferation, inhibition of apoptosis, and cell differentiation that confer positive effects on organ growth and recovery from injury [32]. The proliferative and developmental effects of growth hormones (GHs) on multiple organ systems, including the thymus and the peripheral immune system, are primarily mediated through IGF-1 [33]. IGF-1 has been shown to promote hematopoiesis, [33], prolong T lymphocyte survival [34], and modulate T-cell signaling [35]. IGF-1 receptor (IGF-1R) is expressed on both thymocytes and TECs, and IGF-1 enhances thymopoiesis predominantly through TEC expansion [36]. Moreover, IGF-1 has a beneficial effect on the proliferation and survival of TECs [2,36]. Woo et al. observed evidence of thymic cortical regeneration, replenishment of the peripheral T-cell pool, and enhanced thymus function after rhIGF-I treatment [37]. Therefore, IGF-I is regarded as an important thymotrophic agent that can be used therapeutically to protect or promote thymus function.

BMP-4 is a critical factor during the development of multiple organs, including the thymus where it can target both stromal and hematopoietic compartments [38]. The roles for BMP-4 during thymus organogenesis [39], in the induction of TEC-like cells from pluripotent stem cells [40], and in the maintenance of TEC progenitor [41] are clearly defined. BMP-4 is critical for promoting TEC regeneration after injury [42]. Although it has been known that the majority of BMP-4 production in the adult thymus is derived from fibroblasts and endothelial cells, it was discovered that increased BMP-4 expression is increased with age in TECs rather than fibroblasts and endothelial cells, suggesting that an increase in autocrine BMP-4 signaling in aging TECs may also modulate TEC precursor differentiation [41,43]. Furthermore, BMP-4 has an essential role in thymus regeneration by stimulating TECs to increase the expression of FoxN1, a master regulator in the development, maintenance, and regeneration of TECs, which activates its downstream targets, such as Dll4, a key mediator of thymocyte development and regeneration [38]. These studies suggest the importance of BMP-4 in thymus regeneration and offer a potential clinical approach to improve T-cell immunity.

VEGF-A, a major regulator of angiogenesis and vasculogenesis, is synthesized by TECs and plays a role not only in supporting vascular homeostasis but also in thymus regeneration after involution caused by chemotherapy, radiotherapy, or hormonal therapy [44].

Second, we demonstrated that FCPs significantly promote the gene expression levels of IL-7, IL-21, IL-22R, RANKL, RANK, LTβ, and LTβR, all of which are crucial thymopoietic cytokines and serve as important mediators of T-cell thymopoietic processes during both prenatal T-cell development and postnatal thymus regeneration [45].

IL-7, produced chiefly by TECs, is a cytokine essential for the survival and proliferation of thymocytes and in the differentiation of positively selected CD8^+^ T cells in the thymus [46,47]. IL-7 has been shown to stimulate the proliferation of double-negative (DN) thymocytes, rearrangement of the TCRβ gene, and development of γδ T cells [48]. In the periphery, IL-7 also regulates T-cell homeostasis by enhancing the survival and proliferation of naïve and memory T cells [49]. The interaction of TECs with thymocytes is weakened by the reduced IL-7 expression in aged TECs, resulting in impaired thymopoiesis [2,6,50]. Due to its crucial role in thymopoiesis, T-cell reconstitution, and thymic regeneration, IL-7 has been extensively investigated for its ability to aid recovery following thymus damage from a variety of insults [5,48]. Numerous studies have shown that the administration of IL-7 exogenously can enhance thymopoiesis and T-cell reconstitution following T-cell depletion due to multiple factors, such as lymphoablative therapy, infections, chemotherapy, and radiotherapy, highlighting its use as a potential treatment for numerous clinical situations associated with T-cell immunodeficiency [5,48].

IL-21, a member of the T-cell growth factor family, contributes to allograft rejection and the development of autoimmune and chronic inflammatory diseases by promoting the activation, proliferation, and infiltration of immune cells within the target organ [51]. IL-21 may be involved in the co-stimulation of naïve, but not memory, T cells [52]. IL-21, produced by TECs, can not only enhance thymic function in young and aged mice primarily by its action on double-positive thymocytes, which express high levels of IL-21 receptor after glucocorticoid-induced thymic atrophy, but also facilitate thymic regeneration and the reconstitution of the peripheral T-cell compartment in different models of thymus damage, including glucocorticoid-induced thymic atrophy, aging, and hematopoietic stem cell transplantation [53].

IL-22, a central regulator of epithelial homeostasis, is produced by different immune cell subsets, including CD4^+^ T cells [54]. IL-22R1, RANKL, RANK, LTβ, and LTβR, which are expressed on TECs and found to be upregulated during the early phase of thymic regeneration [45]. IL-22R, a member of the IL-10 cytokine family, is a heterodimer of IL-22R1 (IL-22Rα1, IL-22RA1, also known as IL-22R) and IL-10R2 (IL-10Rβ) [55]. IL-22 first binds to its high-affinity receptor, IL-22R1, which causes a conformational change in IL-22, thereby allowing the IL-22 and IL-22R1 complex to bind to IL-10R2 [55]. IL-22R1 has been recognized as a marker of TEC maturation [6,56]. IL-22 was found to exert a crucial role in the regeneration of the irradiated mouse thymus by directly promoting the proliferation and survival of TECs [2,56].

The tumor necrosis factor receptor superfamily (TNFRSF) member RANK and its cognate ligand RANKL, a member of the TNF superfamily, play not only important roles in lymphocyte development, lymph node organogenesis, and various immune system functions, but also a privileged role in the expansion and differentiation of TECs [57,58]. RANKL is a critical regulator of early thymocyte development, specifically at the stage of pre-TCR expression [57]. The RANK–RANKL axis activates the NF-κB signaling pathways that control the development of Aire^+^ TECs [45,59]. Notably, in our previous studies, we found that RANKL stimulates the proliferation, adhesiveness to thymocytes, and IL-7 expression of TECs, and RANKL expression was strongly upregulated in TECs during thymic regeneration, implying that RANKL could play a role in the development of T cells during thymic regeneration [60,61].

LTβ (LTα1β2) and its unique receptor LTβR play a crucial role in the establishment and regulation of the immune system by facilitating communication between lymphocytes and stromal cells, which is essential for the proper functioning of the immune system. They are linked to the control of the lymphatic migration of immune cells [62]. It was discovered that (1) mice without LTβR have a significantly decreased number of early T-cell progenitors (ETPs), (2) LTβR is required in TECs based on thymus transplant and BM chimera experiments, (3) LTβR regulates the expression of VCAM-1 and ICAM-1, which are important for the thymus entry of ETPs in TECs, and (4) LTβR is necessary for thymic recovery after bone marrow transplantation based on the results that treatment with an agonistic anti-LTβR antibody can improve donor-derived T-cell reconstitution [63]. These results indicate important roles for LTβR signaling in the thymic function that ensures the thymic homing of T-cell progenitors, the trafficking and egress of thymocytes, the differentiation of TECs, and the generation of a diverse and self-tolerant T-cell repertoire [62]. Recently, the importance of the LTβ/LTβR axis in controlling the recovery of the thymic function after a myeloablative conditioning regimen has also been proposed, opening novel perspectives in the repertoire of regenerative medicine [62].

Third, we demonstrated that FCPs significantly promote the gene expression levels of SDF-1, CCL21, CCL25, and CXCL5, all of which are pivotal thymopoietic chemokines that serve as important mediators of thymopoietic processes in both prenatal and postnatal T-cell generation.

SDF-1 (also known as CXC motif chemokine 12, CXCL12), a member of the chemokine CXC subfamily and expressed in TECs, is a potent chemoattractant for early thymocytes as well as T lymphocytes, and SDF-1/CXCR4 signaling also plays a key role in the migration, survival, expansion, and subsequent differentiation of human ETPs and T-cell development [42,64]. SDF-1 could be used to therapeutically promote the reconstitution of T-cell immunity by enhancing thymopoiesis in some of those pathologic situations associated with loss or damage to the peripheral T-cell pool [65].

CCL21 (also known as 6Ckine, Exodus-2, and thymus-derived chemotactic agent-4), a CC family chemokine, has the function of chemotactic cell migration of a variety of cells, especially on lymphocytes [66]. It was recently identified that CCL21 is involved in the establishment of central self-tolerance in T cells and is an important regulator of newly selected αβ T-cell emigration from the thymus, which is a key mechanism in establishing a functional adaptive immune system [67,68]. CCL25 (also known as thymus-expressed chemokine, TECK), a CC chemokine that regulates the trafficking of lymphocytes in the thymus and peripheral organs, is found in both the cortical epithelial cells and dendritic cells of the thymus [69]. CCL25, along with SDF-1 and CCL21, guides the homing of T-cell progenitors into the thymus and contributes to driving T-cell development [70,71]. CXCL5, produced by a variety of cells, including epithelial cells, is linked to immune cell recruitment. It has been shown that the expression levels of NF-κB, MAPK, and ICAM-1 were reduced in smoke-induced lung inflammation in CXCL5-deficient mice [72].

Zubkova et al. observed an increase in the expression of IL-7, SDF-1, and CCL25 mRNA in the involuted thymus of mice after administering dexamethasone and estrogen or exposure to radiation, suggesting that the upregulation of these mRNAs reflects the progression of thymic regeneration [73]. In our previous study, we presented a new insight into the role of IL-7, SDF-1, and CCL25 by nerve growth factor in the promotion of thymopoiesis [74].

Fourth, we demonstrated that FCPs significantly promote the gene expression levels of the cell signaling molecules Dll1, Dll4, and Wnt4, which are the key players critical for interactions between T-cell precursors and TECs, including the specification, commitment, and development of thymocytes [75].

T cells develop in the thymus based on signaling from multiple stromal cell types, particularly TECs. The Notch signaling pathway plays an essential role in a wide variety of biological processes, including the determination of T-cell fate from hematopoietic progenitor cells (HPCs) [76]. In the absence of Notch signaling, the thymus becomes a place where the development of B cells instead of T cells is supported [77]. The Notch ligands Dll1 and Dll4 in TECs function as proper ligands to provide HPCs with Notch signals required for T-cell specification in the thymus, thereby supporting T lymphopoiesis [75]. In addition, Dll4 controls the maturation of T-cell progenitors to the CD4^+^CD8^+^ stage [75]. Particularly, Dll4 is critically important for steady-state thymic function, as Dll4 deletion leads to the complete abrogation of T-cell development, and the concentration of intrathymic Dll4 expression can profoundly impact T-cell development and thymus size, indicating the important role of Dll4 in the regulation of thymic regeneration [75]. Wnt4 was found to play an important role in thymic recovery after hematopoietic stem cell transplantation, suggesting that Wnt4 regulates the number of TECs as well as thymocytes in the prenatal and postnatal thymus [78].

Fifth, we demonstrated that FCPs significantly promote the gene expression levels of the co-stimulatory molecules CD40, CD80 (also known as B7-1), and CD86 (also known as B7-2), which are expressed on the surface of TECs and provide a critical signal for T-cell activation, together with the TCR and antigenic peptide-loaded major histocompatibility complex (MHC) interaction, thereby influencing T-cell differentiation and fate. Regulated co-stimulation in the thymus is also crucial for T-cell development [79]. It was shown that CD28–CD80/86 and CD40–CD40L co-stimulatory interactions mediate the negative selection and self-tolerance of T cells, upregulate the expression of LTα, LTβ, and RANK in the thymus, and are necessary for the development of thymocytes and TECs [80]. Without CD28–CD80/86 and CD40–CD40L signaling, the tolerance-inducing thymic medullary compartment fails to properly develop and the SP thymocytes become autoreactive [80].

Sixth, we demonstrated that FCPs significantly promote the gene expression levels of the cell adhesion molecules ICAM-1 and VCAM-1, which are highly expressed by TECs, play a central role in the binding interactions between TECs and thymocytes, and also act as co-stimulatory molecules in thymocyte positive selection [81]. Notably, in our previous studies, we showed that the expression of RANKL is increased during thymic regeneration, and RANKL stimulates the expression of ICAM-1 and VCAM-1 by TECs [60,61]. Taken together, FCPs both directly and indirectly increase the expression of ICAM-1 and VCAM-1 in TECs, leading to the promotion of thymic regeneration.

Seventh, we demonstrated that FCPs significantly promote the gene expression levels of FoxN1, a TEC-specific transcription factor, which is a master regulator of TEC differentiation, growth, and function as well as thymus development [82]. Moreover, FoxN1 regulates the key target genes essential for T-cell development in postnatal TECs and enables TECs to support thymopoiesis and T-cell development [82,83]. The reduced expression of FoxN1 was found to contribute to age-related thymic involution [84]. Furthermore, it has been shown that the forced induction of FoxN1 is capable of reversing age-related thymic atrophy, and the recombinant FoxN1 protein can enhance T-cell reconstitution after hematopoietic stem cell transplantation, indicating that FoxN1 plays an important role in thymic regeneration [2,85]. Importantly, SDF-1, CCL25, Dll4, and stem cell factor (SCF, also known as c-kit ligand) have been identified as the four direct targets of FoxN1 [86].

In addition, the exogenous administration or forced expression of FoxN1, Dll4, FGF-7, IL-7, SDF-1, and SCF has been described to promote thymic regeneration [5]. Consistent with this finding, our study demonstrated that FCPs triggered the gene expression of FoxN1 as well as FGF-7, IL-7, SDF-1, CCL25, and Dll4, strongly indicating that FCPs could be a promising therapeutical approach to promote thymopoiesis and the reconstitution of T-cell immunity in numerous clinical situations associated with T-cell deficiency.

Eighth, we demonstrated that FCPs significantly promote the gene expression levels of leptin, which is one of the best-known hormone markers for obesity and exerts an important role in both prenatal and postnatal T-cell development in the thymus [87]. Leptin, an adipokine and neuroendocrine hormone closely linked to obesity, regulates not only adipogenesis and energy balance but also functioning of the thymus [88]. The leptin–thymus axis is relevant to the reduced thymic function with aging [89], and decreased activity of this axis results in thymic involution [87]. Accumulating evidence has suggested that a decreased level of leptin in aged individuals further perpetuates the age-dependent thymic involution accompanied by a concomitant loss of Foxn1-inducing signals, such as Wnt4 or BMP-4 in the aged thymus, indicating that leptin plays an important role in thymic regeneration [2].

Ninth, we demonstrated that FCPs significantly promote the gene expression level of cathepsin L, which is a critical protease for the MHC class II antigen processing and presentation pathway that plays an essential role in the interactions of TCRs of developing thymocytes with MHC/peptide ligands through invariant (Ii, CD74) chain degradation in cortical TECs [90]. Cathepsin L, a cortical TEC-expressed protease, is also necessary for CD4^+^ positive selection by playing a part in the presentation of antigenic peptides within MHC molecules to developing thymocytes [2,91]. Together, these results along with our finding that the level of cathepsin L was increased in both normal and injured TECs after treatment with FCPs provide evidence for the role of FCPs in the potentiation of the functional activity of TECs.

Tenth, we demonstrated that FCPs significantly promote the gene expression levels of CK5 and CK8, intermediate filaments that are a unique characteristic of TECs among the various thymic cell populations, and form tonofilaments in the cytoplasm of TECs to maintain the tensile strength and integrity of TECs and their network, thymic epithelial reticulum, creating the cellular framework of the thymic stromal cellular microenvironment [92]. Notably, we previously showed that the expression of CK5, CK8, and CK14 increases in TECs during mouse thymus regeneration after CP-induced acute thymic involution, suggesting that CKs in TECs are correlated with the stage of TEC development and differentiation and the functional states of TECs [92]. Thus, the increased level of CK5 and CK8 in both normal and injured TECs after treatment with FCPs observed in this study indicates the augmented activity of TECs induced by FCPs.

The NF-κB transcription factor plays a key role in the regulation of the maturation and functional activity of TECs as well as the development of the thymus [4,21,22]. NF-ĸB was also found to be related to the proliferation, adhesion, and migration of NIH3T3 cells [93]. In addition, the NF-ĸB signaling pathway has a close association with epithelial wound healing [93,94]. The adhesion experiments in this study revealed that FCPs increase the number of adherent thymocytes with TECs, which is accompanied by the increased expression of ICAM-1 and VCAM-1 in TECs, which is a crucial mechanism for thymocyte–TEC interactions that constitute important events in T-cell development through the activation of the NF-ĸB signaling pathway [61,74,95,96]. It is notable that both NF-ĸB and Wnt4 are associated with T-cell development [97,98]. However, it was discovered that they could act in a very different way. NF-ĸB plays a key role in regulating T-cell functions and development, while Wnt4 is involved in the regulation of age-related thymic involution [97,98]. Although the precise molecular mechanisms that underlie the relationship between NF-kB and Wnt4 in T-cell development are not yet fully understood, putative NF-kB/Rel binding sites have been identified at the Wnt4 enhancer region, suggesting that NF-kB signaling may directly regulate Wnt4 expression as an upstream regulator [99]. Taken together, these results, along with the effects of FCPs on cell proliferation and thymopoietic factor expression in TECs, provide evidence for a pivotal role of FCPs in T-cell differentiation and regeneration via the NF-ĸB signaling pathway.

Furthermore, our in vivo experiments illustrate that FCPs prevented the decline in the weights of the thymus and body as well as the thymus index induced by CP. In addition, the results of HE staining in the thymus and spleen as well as the flow cytometric analysis of the CD4^+^ T cell population and the expression of major thymopoietic factors in the thymus, including FGF-7, IL-7, IL-21, SDF-1, CCL21, CXCL5, ICAM-1, and FoxN1, also prove that FCPs could prevent the damage to the thymus and spleen induced by CP. These results are consistent with our previous study, where we demonstrated that FCPs have a cytoprotective role in TECs against cisplatin-induced cytotoxicity [20].

Our study underscores notable advancements yet harbors a potential limitation requiring acknowledgment. While we unveiled significant mRNA expression modifications that elucidated the effects of FCPs on the thymopoietic activities of TECs as well as thymic regeneration, the conclusive translation to protein expression and ultimate functionality remains to be authenticated. Thus, subsequent research must meticulously explore and validate the observed mRNA changes at the protein level, ensuring a holistic, mechanistic comprehension of the stimulative and regenerative properties of FCPs in the thymus.

Overall, our findings indicate that FCPs have the potential to be utilized as a therapeutic strategy to facilitate the protection of TECs, reconstitution of T cells, and regeneration of the thymus, thereby counteracting the impact of immunological insults and immunosuppression on immune organs. Moreover, FCPs may serve as a powerful natural agent for modulating the immune system and could be considered for clinical use in immunocompromised patients caused by various factors, including chemotherapy.

## 4. Materials and Methods

### 4.1. Cell Culture and Reagents

The mouse TECs used in this study were obtained from Dr. Barbara B. Knowles at The Jackson Laboratory in Bar Harbor, ME, USA. The HepG2 cells, which are a human hepatoblastoma cell line, were acquired from the American Type Culture Collection (ATCC) located in Rockville, MD, USA. The cells were cultured in Dulbecco’s modified Eagle’s medium (DMEM; Hyclone, GE Healthcare Life Sciences, Logan, UT, USA), which was supplemented with 10% fetal bovine serum (FBS), 100 IU/mL penicillin, and 100 mg/mL streptomycin (all from Gibco, Thermo Fisher Scientific, Waltham, MA, USA). The culture was maintained in a humidified atmosphere containing 5% CO_2_ at a temperature of 37 °C. The subconfluent cells were collected using trypsin-EDTA and subsequently utilized for further experimental procedures. The media were replaced on a bi-daily basis.

CP, Bay 11-7082, Bay 11-7085, 4′,6-diamidino-2-phenylindole (DAPI), and bicinchoninic acid (BCA) were purchased from Sigma-Aldrich, located in St. Louis, MO, USA. The antibodies targeting NF-κB p50 and Ki-67 were acquired from Santa Cruz Biotechnology (Santa Cruz, CA, USA) and Invitrogen (Carlsbad, CA, USA), respectively. The FCPs derived from tilapia specimens were obtained from Geltech, a company based in Busan, Republic of Korea. Detailed information on the physicochemical characteristics of these FCP samples may be found in our earlier publication [100]. All additional reagents and substances utilized in the experiment were provided by Sigma-Aldrich.

### 4.2. Cell Proliferation Assay

The TECs were seeded at a density of 8 × 10^3^ cells per well on 96-well flat-bottom culture plates (SPL Life Sciences, Pocheon, Republic of Korea). The cells were then treated with the specified doses of FCPs for a duration of 48 h, either in the presence or absence of Bay 11-7082 or Bay 11-7085 for 15 or 30 min. The assessment of cell proliferation was conducted using the colorimetric WST-1 conversion assay, namely the EZ-Cytox assay kit provided by Daeil Lab Service in Seoul, Republic of Korea. In each well, a 10 μL volume of WST-1 reagent was introduced, followed by incubation of the cells for a duration of 2 h. This incubation was carried out in a humidified incubator at a temperature of 37 °C, with a CO_2_ concentration of 5%. The measurement of the absorbance of the formazan dye, which is produced through the reaction between dehydrogenase and WST-1 in metabolically active cells, was conducted using a microplate reader (Tecan, Männedorf, Switzerland) at a wavelength of 450 nm, following the guidelines provided by the manufacturer. Subsequently, the percentage of cell proliferation was determined through calculations. The experiments were conducted in triplicate.

### 4.3. Evaluation of Immunofluorescence Using Confocal Microscopy

TECs (1 × 10^4^ cells/well) were cultivated in 8-well glass slides (SPL Life Sciences) for the purpose of conducting an immunofluorescence analysis. The objective of this research was to assess the nuclear translocation of NF-κB p50 in TECs. Prior to the analysis, the TECs were treated with 0.08% FCP for a duration of 24 h. Following this treatment, the TECs were further treated with 5 μM Bay 11-7082 for a period of 15 min. The monolayer cells were treated for 10 min with a 4% paraformaldehyde solution in phosphate-buffered saline (PBS) for fixation. Subsequently, the cells were permeabilized using a 0.1% Triton X-100 solution in PBS for a period of 10 min. The cells were subjected to incubation with a monoclonal antibody against NF-κB p50 (Invitrogen) at a dilution of 1:200 and maintained at 4 °C overnight. Following washing in PBS, the samples were subjected to an incubation period of 1 h at room temperature (RT). During this incubation, the samples were treated with Alexa Fluor 594 chicken anti-rat IgG (H+L) secondary antibody (Invitrogen) at a dilution of 1:1000. The immunofluorescence analysis was conducted utilizing a laser confocal microscope (LSM900; Carl Zeiss, Jena, Germany).

### 4.4. RNA Isolation and cDNA Synthesis

The isolation of total RNA from cells was performed using TRIzol reagent, manufactured by Favorgen Biotech Corp located in Pingtung, Taiwan. The determination of RNA quality and quantity was conducted by a NanoDrop 2000 spectrophotometer manufactured by Thermo Scientific, located in Waltham, MA, USA. The synthesis of first-strand cDNA was performed using 1 µg of total RNA from each sample, employing the HiSenScript™ RH(−) RTase cDNA Synthesis Kit (iNtRON Biotechnology, Sungnam, Republic of Korea). The reaction mixture was heated to 85 °C for 10 min to stop the reaction after being incubated at 45 °C for 1 h.

### 4.5. Quantitative Real-Time PCR (qRT-PCR)

The qRT-PCR experiment was conducted using the CFX Connect Real-Time equipment (Bio-Rad, Hercules, CA, USA) and the SsoAdvanced Universal SYBR Green Supermix (Bio-Rad) with the DNA-binding dye SYBR Green I. The particular primers utilized for qRT-PCR are outlined in Table S1. The amplification process was performed in triplicate for all samples. The quantification of gene expression levels was performed via the 2^−ΔΔCt^ methodology, with normalization to the expression levels of glyceraldehyde-3-phosphate dehydrogenase (GAPDH). The control sample was standardized to a value of 1, and the relative expression of the other samples was subsequently determined based on this standardization.

### 4.6. Quantitation of Thymocyte Adhesion to TECs

TEC-thymocyte adhesion assay was performed as previously established [101]. Briefly, 5 × 10^5^ TECs were seeded in 6-well plates and were treated with 0.08% FCP in serum-free (SF) DMEM for 24 h. The media were replaced with SF DMEM containing 5 μM Bay 11-7082 or Bay 11-7085 for 2, 5, or 7 min prior to seeding 1 × 10^7^ freshly isolated thymocytes at onto the layer of pretreated TECs. After 24 h of co-culture, the cells were gently washed for the removal of non-adherent thymocytes, and 0.5 mM EDTA (Sigma-Aldrich) in PBS was used to collect the adherent thymocytes. Collected thymocytes were stained with trypan blue and counted using a hemocytometer.

### 4.7. Flow Cytometry

For the assessment of TEC proliferation, 1 × 10^6^ cells/tube were collected, fixed using the Cytofix/Cytoperm solution (BD Biosciences, San Jose, CA, USA), and then permeabilized using the Perm/Wash solution (BD Biosciences). The cells were washed and then stained with Ki-67 (1:100; Invitrogen) for 1 h followed by anti-rat Alexa Fluor 594-conjugated secondary antibody (1:100; Invitrogen) for 30 min at RT. Samples were analyzed on a FACS Canto-II flow cytometer (BD Biosciences), and data were processed using FlowJo 10.3.0 (Tree Star; Ashland, OR, USA).

### 4.8. In Vitro TEC Treatment of CP

**Figure 8:**
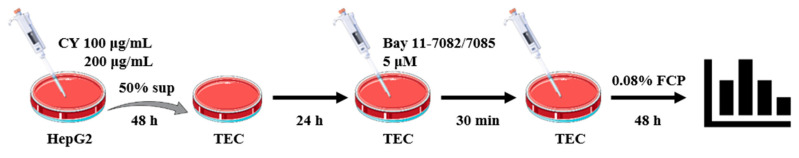
Schematic diagram of the in vitro TEC experimental procedures. Parts of the figure were drawn by using pictures from Servier Medical Art. Servier Medical Art by Servier is licensed under a Creative Commons Attribution 3.0 Unported License “https://creativecommons.org/licenses/by/3.0/ (accessed on 7 March 2023)”.

To explore the role of FCPs in the protection of TECs against CP-induced cytotoxicity, we conducted experiments that are schematically depicted in Figure 8. Briefly, we used the human HepG2 cell line for the production of active metabolites of CP since CP is a prodrug that must be metabolically activated by the hepatic enzyme cytochrome P450 enzymes. Therefore, HepG2 cells (8 × 10^5^ cells/well) were treated with 100 μg/mL or 200 μg/mL of CP, and after 48 h, the conditioned media derived from CP-treated HepG2 cells (CM-CP) were collected and centrifuged at 5000 rpm for 5 min. The CM-CP was filtered using 0.2 µm membrane filters and stored at −20 °C. Subsequently, TECs (8 × 10^5^ cells/well) were treated with a 1:1 mixture of CM-CP and DMEM supplemented with 10% FBS, 100 IU/mL penicillin, and 100 mg/mL streptomycin for 24 h. After the cells were washed, they were treated with 5 μM Bay 11-7082 or Bay 11-7085 for 30 min prior to FCP treatment for 48 h.

### 4.9. Animals and Experimental Design

**Figure 9:**
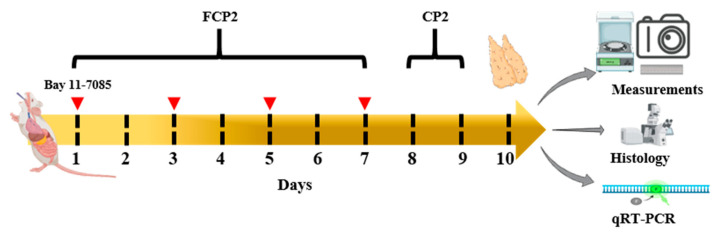
Schematic diagram of the in vivo experimental procedures. Parts of the figure were drawn by using pictures from Servier Medical Art. Servier Medical Art by Servier is licensed under a Creative Commons Attribution 3.0 Unported License “https://creativecommons.org/licenses/by/3.0/ (accessed on 7 March 2023)”.

To explore the role of FCPs in the regeneration after CP-induced thymus degeneration, we conducted experiments that are schematically depicted in Figure 9. Briefly, five-week-old male C57BL/6 mice purchased from Dae Han Bio Link (Seoul, Republic of Korea) were housed in a specific pathogen-free animal room under a 12 h light/12 h dark cycle and were fed a normal diet and water given ad libitum. After one week of the adaptation period, the mice were randomly divided into designated groups consisting of 4 mice each. For histomorphological analysis, in the NC1 group, the mice were administered a 0.2 mL sterile physiological saline solution intragastrically (i.g.) for 7 days once daily. In the CP1 group, the mice were intraperitoneally (i.p.) injected with a single dose of 450 mg/kg CP on day 1. In the FCP1 group, the CP-pretreated mice were administered i.g. with 200 mg/kg/day FCP dissolved in the physiological saline solution for 7 consecutive days from day 1.

For further analysis, in the NC2 group, the mice were administered a 0.2 mL sterile physiological saline solution i.g. for 7 days once daily. In the CP2 group, the mice were injected with 450 mg/kg/day CP i.p. on days 8 and 9. In the FCP2 group, the mice were administered with 200 mg/kg/day FCP dissolved in the physiological saline solution i.g. 7 consecutive days from day 1. In the FCP2+CP2 group, the mice were i.p. injected with 450 mg/kg/day CP on days 8 and 9 after the mice were administered 200 mg/kg/day FCP i.g. for 7 consecutive days from day 1. In the FCP2+CP2+Bay group, the mice were i.p. injected with 10 mg/kg/day Bay 11-7085 on designated days along with the administration of 200 mg/kg/day FCP i.g. for 7 consecutive days from day 1 as well as the injection of 450 mg/kg/day CP i.p. on days 8 and 9. The mouse body weights were recorded throughout the entire study period before the daily administration. At the 24 h time point following the last dose, the animals were weighed and then euthanized using the gradual-fill method of CO_2_ euthanasia. The experimenters were blinded to the treatment of mice while processing data. All procedures were approved by Pusan National University-Institutional Animal Care and Use Committee (PNU-IACUC) on 20 July 2023 and carried out under the IACUC-approved protocol (approval number PNU-2023-0203). All sections of this report adhere to the ARRIVE Guidelines for reporting animal research [102]. A complete ARRIVE guidelines checklist is included in Checklist S1.

### 4.10. HE Staining

Each freshly removed mouse thymus was embedded in an optimal cutting temperature (OCT) compound and frozen with liquid nitrogen. Tissue sections (5 µm thick) were cut on a Reichert cryostat (Leica, Deerfield, IL, USA) and placed on 3-aminopropyltriethoxysilane-coated slides. After being dried, the cryosections were fixed in acetone for 10 min at −20 °C and stained with hematoxylin and eosin.

### 4.11. Effect of FCPs on Mouse Thymus Index

At the 24 h time point after the last CP treatment, the weights of mice and thymuses were used to calculate the immune organ indices according to the following formula:

Index (mg/g) = weight of thymus/body weight

### 4.12. Immunofluorescence Analysis of CD4 Expressions in the Thymus

Each freshly isolated thymus embedded in the OCT compound was frozen with liquid nitrogen and cut into 5 µm slices. The sections were incubated at 4 °C overnight with anti-mouse CD4 monoclonal antibodies (Biolegend, San Diego, CA, USA). The sections were then incubated with an affinity-purified F(ab’)_2_ fragment donkey anti-rabbit FITC-conjugated antibody (1:100, Jackson Immuno Research Laboratories, West Grove, PA, USA). The labeled cells were examined with an Olympus BX61 microscope (Olympus, Tokyo, Japan) equipped with a CCD camera and fluorescence intensities were analyzed using ImageJ (version 1.53; National Institutes of Health, Bethseda, MD, USA). AnalySIS software.

### 4.13. Statistical Analysis

All quantitative results were determined by the unpaired two-tailed Student’s *t*-test and expressed as the mean ± standard deviation (SD) of at least three independent experiments. Values of *p* < 0.05 were considered statistically significant.

## 5. Conclusions

The findings of the present study demonstrate for the first time that FCPs stimulate activities of TECs, including cell proliferation, thymocyte adhesion to TECs, and the gene expression of thymopoietic factors such as FGF-7, IGF-1, BMP-4, VEGF-A, IL-7, IL-21, RANKL, LTβ, IL-22R, RANK, LTβR, SDF-1, CCL21, CCL25, CXCL5, Dll1, Dll4, Wnt4, CD40, CD80, CD86, ICAM-1, VCAM-1, FoxN1, leptin, cathepsin L, CK5, and CK8 through the NF-κB signaling pathway. In addition, we revealed not only the protective effects of FCPs on TECs against CP-induced cellular injury but also the promoting effects of FCPs on thymus regeneration after the CP-induced thymic degeneration via the NF-κB pathway. Therefore, these results suggest that FCPs may be a promising protective agent in TEC injury induced by cytotoxicity as well as an auspicious regenerative agent to enhance thymopoiesis in several clinical situations associated with T-cell deficiency. Furthermore, the data of the present study may provide new insights into the therapeutic approach for the future application of FCPs in the prevention and treatment of a variety of cytotoxic stress-mediated injuries in TECs as well as acute or age-related thymic involution.

## Data Availability

Not applicable.

## Supplementary Materials

The following supporting information can be downloaded at: https://www.mdpi.com/article/10.3390/md21100531/s1, Figure S1: FCPs stimulate thymopoietic responses through NF-ĸB signaling pathway in TECs as detected using qRT-PCR after treatment with Bay 11-7085. Table S1: The list of primers used in the current study.
