# Supplementary material for: Fish Collagen Peptides Enhance Thymopoietic Gene Expression, Cell Proliferation, Thymocyte Adherence, and Cytoprotection in Thymic Epithelial Cells via Activation of the Nuclear Factor-κB Pathway, Leading to Thymus Regeneration after Cyclophosphamide-Induced Injury
Source: Mar Drugs. 2023 Oct 12;21(10):531. doi: 10.3390/md21100531 (PMC10608061; doi:10.3390/md21100531)
Supplement: Supplementary file 1 [file marinedrugs-21-00531-s001.zip › Table S1.pdf]

Gene name	Forward (5′-3′)	Reverse (5′-3′)
BMP-4	GCCGAGCCAACACTGTGAGGA	GATGCTGCTGAGGTTGAAGAGG
Cathepsin L	GGAAAATGGAGGTCTGGACTCG	GTGTCATTAGCCACAGCGAACTC
CCL21	GGGTCAGGACTGCTGCCTTAAG	AGCTCAGGCTTAGAGTGCTTCC
CCL25	AAGGCTAGTCCACTGGAAGAGC	GTGGCACTCCTCACGCTTGTAC
CD40	ACCAGCAAGGATTGCGAGGCAT	GGATGACAGACGGTATCAGTGG
CD80	CCTCAAGTTTCCATGTCCAAGGC	GAGGAGAGTTGTAACGGCAAGG
CD86	ACGTATTGGAAGGAGATTACAGCT	TCTGTCAGCGTTACTATCCCGC
CK5	GAACAGAGGCTGAGTCCTGGTA	TCTCAGCCTCTGGATCATTCGG
CK8	TGGAAGGACTGACCGACGAGAT	GGCACGAACTTCAGCGATGATG
CXCL5	CCGCTGGCATTTCTGTTGCTGT	CAGGGATCACCTCCAAATTAGCG
Dll1	GCTGGAAGTAGATGAGTGTGCTC	CACAGACCTTGCCATAGAAGCC
Dll4	GGGTCCAGTTATGCCTGCGAAT	TTCGGCTTGGACCTCTGTTCAG
FGF-7	TGTTCTGTCGCACCCAGTGGTA	TTCCAACTGCCACGGTCCTGAT
FoxN1	CGGAAGCCTTCCAGTCAGTGAA	TGTTGAGGGACAGGTTATGGCG
ICAM-1	AAACCAGACCCTGGAACTGCAC	GCCTGGCATTTCAGAGTCTGCT
IGF-1	GTGGATGCTCTTCAGTTCGTGTG	TCCAGTCTCCTCAGATCACAGC
IL-21	GCCTCCTGATTAGACTTCGTCAC	CAGGCAAAAGCTGCATGCTCAC
IL22R	TTTCCTCGTCGGCTTGCTCTGT	CGTGTTCTTGGATGAAGCGTAGG
IL-7	CAGGAACTGATAGTAATTGCCCG	CTTCAACTTGCGAGCAGCACGA
Leptin	GCAGTGCCTATCCAGAAAGTCC	GGAATGAAGTCCAAGCCAGTGAC
LTβ	CCTGTTGTTGGCAGTGCCTATC	GACGGTTTGCTGTCATCCAGTC
LTβR	TCCTTGGAGGAAGTGGTGCTAC	CGGTCACATGAATGCCATTCGC
RANK	GGACAACGGAATCAGATGTGGTC	CCACAGAGATGAAGAGGAGCAG
RANKL	GTGAAGACACACTACCTGACTCC	GCCACATCCAACCATGAGCCTT
SDF-1	GACTGGCATAGTCGGCAATGGA	CAAAGAGGAGGTCAGCCACTGA
VCAM-1	AAACCAGACCCTGGAACTGCAC	GCCTGGCATTTCAGAGTCTGCT
VEGF-A	CTGCTGTAACGATGAAGCCCTG	GCTGTAGGAAGCTCATCTCTCC
Wnt4	GAGAACTGGAGAAGTGTGGCTG	CTGTGAGAAGGCTACGCCATAG
GAPDH	CATCACTGCCACCCAGAAGACTG	ATGCCAGTGAGCTTCCCGTTCAG
