# Supplementary figures and images for: Fish Collagen Peptides Enhance Thymopoietic Gene Expression, Cell Proliferation, Thymocyte Adherence, and Cytoprotection in Thymic Epithelial Cells via Activation of the Nuclear Factor-κB Pathway, Leading to Thymus Regeneration after Cyclophosphamide-Induced Injury
Source: Mar Drugs. 2023 Oct 12;21(10):531. doi: 10.3390/md21100531 (PMC10608061; doi:10.3390/md21100531)

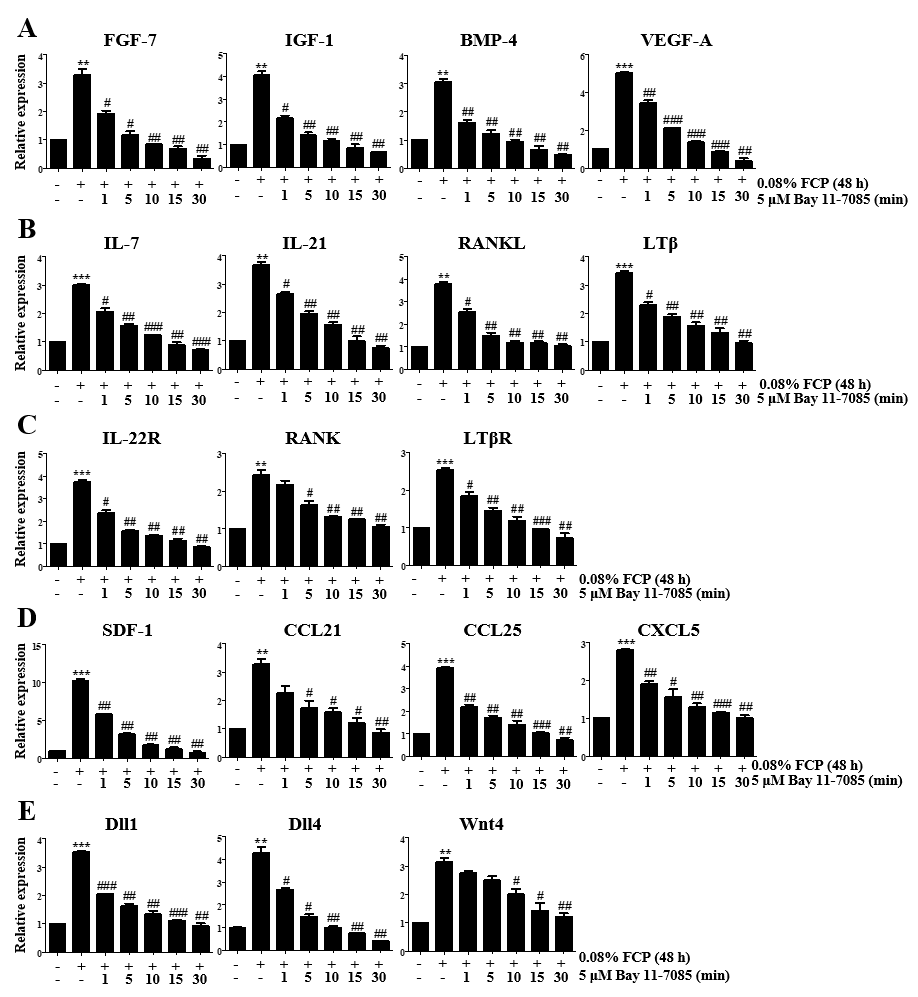

Supplement: Supplementary file 1 [file marinedrugs-21-00531-s001.zip › Figure S1 (1st part).tif]

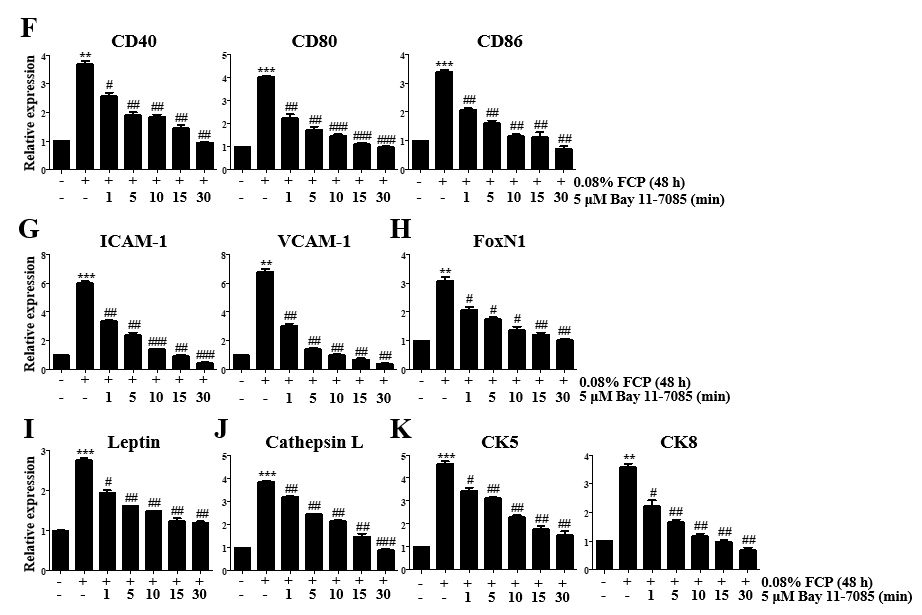

Supplement: Supplementary file 1 [file marinedrugs-21-00531-s001.zip › Figure S1 continued.tif]
